# Epithelial Transport of Immunogenic and Toxic Gliadin Peptides *In Vitro*

**DOI:** 10.1371/journal.pone.0113932

**Published:** 2014-11-21

**Authors:** Christian Zimmermann, Silvia Rudloff, Günter Lochnit, Sevgi Arampatzi, Wolfgang Maison, Klaus-Peter Zimmer

**Affiliations:** 1 Department of Pediatrics, Justus Liebig University Giessen, Feulgenstr. 12, D-35392, Giessen, Germany; 2 Institute of Nutritional Science, Justus Liebig University Giessen, Wilhelmstr. 20, D-35392, Giessen, Germany; 3 Institute of Biochemistry, Medical Faculty, Justus Liebig University Giessen, Friedrichstr. 24, D-35392, Giessen, Germany; 4 Institute of Organic Chemistry, Justus Liebig University Giessen, Heinrich-Buff-Ring 58, D-35392, Giessen, Germany; 5 Pharmaceutical and Medicinal Chemistry, Universität Hamburg, Bundesstr. 45, D-20146, Hamburg, Germany; Consejo Superior de Investigaciones Cientificas, Spain

## Abstract

**Scope:**

Celiac disease is an autoimmune disorder caused by failure of oral tolerance against gluten in genetically predisposed individuals. The epithelial translocation of gluten-derived gliadin peptides is an important pathogenetic step; the underlying mechanisms, however, are poorly understood. Thus, we investigated the degradation and epithelial translocation of two different gliadin peptides, the toxic P31–43 and the immunogenic P56–68. As the size, and hence, the molecular weight of peptides might have an effect on the transport efficiency we chose two peptides of the same, rather short chain length.

**Methods and Results:**

Fluorescence labeled P31–43 and P56–68 were synthesized and studied in a transwell system with human enterocytes. Fluorometric measurements were done to reveal antigen translocation and flow cytometry as well as confocal microscopy were used to investigate cellular uptake of peptides. Structural changes of these peptides were analysed by MALDI-TOF-MS. According to fluorescence intensities, significantly more P31–43 compared to P56–68 was transported through the enterocyte layer after 24 h incubation. In contrast to previous reports, however, mass spectrometric data do not only show a time-dependent cleavage of the immunogenic P56–68, but we observed for the first time the degradation of the toxic peptide P31–43 at the apical side of epithelial cells.

**Conclusion:**

Considering the degradation of gliadin peptides by enterocytes, measurement of fluorescence signals do not completely represent translocated intact gliadin peptides. From our experiments it is obvious that even short peptides can be digested prior to the translocation across the epithelial barrier. Thus, the chain length and the sensibility to degradations of gliadin peptides as well as the integrity of the epithelial barrier seem to be critical for the uptake of gliadin peptides and the subsequent inflammatory immune response.

## Introduction

With a prevalence of 1% celiac disease is one of the most common inflammatory disorders in Western populations; it is mainly triggered and maintained by gluten, storage proteins in wheat, rye, and barley [1], [2]. In addition to gluten as environmental factor, a genetic predisposition is required to develop celiac disease. The vast majority of celiac patients express human leukocyte antigen (HLA)-DQ2 and/or HLA-DQ8 by which immunogenic gluten-derived gliadin peptides (GP) are presented and thereby induce the proliferation of gluten sensitive T-cells [1]. The oral intake of gluten may then lead to an initiation of innate and adaptive immune responses which result in cell damage and villus atrophy in the small intestine with subsequent malabsorption and diarrhoea. Up to now the only treatment is a life-long gluten-free diet not only to enable mucosal recovery but also to minimize the risk for the development of enteropathy-associated T-cell lymphoma which has been seen to be markedly increased in patients with untreated celiac disease [2], [3].

In this context, gliadins as a fraction of gluten proteins [4] were identified as important inflammatory stimuli in celiac patients [5]. Currently, two groups of GP are considered to contribute to the disease process in a different manner. Therefore, a distinction is made between immunogenic and toxic GP [6]. Immunogenic GP contain sequences which stimulate HLA-DQ2 or HLA-DQ8 restricted T-cell clones derived from celiac patients thereby inducing an adaptive immune response [7]–[11]. For example, P56–68 is considered to be a sequence representative of immunogenic peptides located at amino acid position 56–68 of α-gliadin [11]. In contrast, toxic GP were shown to induce non T-cell mediated mucosal damage in biopsies from celiac small intestine [12]–[14]. P31–43, a peptide sequence located at position 31–43 of α-gliadin represents a GP from the group of toxic peptides [12].

As a prerequisite for these immune reactions, GP have to resist proteolytic digestion and to pass the intestinal epithelial barrier and gain access to the *lamina propria*. Dietary proteins are digested by soluble gastric and pancreatic proteases and are subsequently hydrolysed by peptidases located in the brush border membrane of enterocytes [15]. Furthermore, antigens can be degraded in the endocytotic pathway of enterocytes [10]. However, proline-rich peptides are inaccessible for most proteases leading to the hypothesis that GP which are rich in proline are resistant to proteolytic breakdown [16]. These antigens may then cross the epithelial barrier via paracellular pathways regulated by tight junctions which are thought to be accessible for small antigens with a maximal molecular weight of about 5,500 Da [17]. Alternatively, antigens may pass the monolayer transcellular by endocytosis [18].

Up to now, underlying mechanisms of translocation and processing of the GP P31–43 and P56–68 in enterocytes are not clear. Matysiak-Budnik et al. [19] reported that the immunogenic P57–68 was partially degraded by brush-border peptidases in biopsies from control but not in those from celiac patients. During the transport process across the epithelial barrier, however, it was completely degraded in biopsies from both, control and celiac patients. In contrast, the toxic P31–49 was shown to be resistant against degradation by brush-border peptidases in celiac and control patients. Still, the intact peptide was only able to pass the epithelial barrier in biopsies from celiac patients but not in those from controls. Thus, the specific ways of epithelial translocation of immunogenic and toxic GP are considered to be a crucial step in the pathogenesis of celiac disease.

Using fluorescence labeled immunogenic and toxic GP in enterocytes and investigating their intracellular transport, it has been suggested that the immunogenic P56–68 was transported into late endosomes whereas the toxic P31–43 and P31–49 remained in early endosomes. Due to the transport of P56–68 into HLA-class-II positive late endosomes a degradation and/or a presentation by enterocytes was hypothesized. In contrast, it is supposed that toxic GP stay intact on the endocytotic route and are released at the basolateral side of the enterocyte [20], [21].

In the present study, we investigated the proteolytic breakdown and transport of two short GP of the same chain length, the toxic P31–43 and the immunogenic P56–68 by enterocytes in a human intestinal cell line (Caco-2) as a model system for transport in gut epithelium [22], [23]. In this context, a major question was whether fluorescence signals alone, without identifying the underlying structures, can be used for an unequivocal conclusion regarding the endocytosis and translocation of GP.

## Material and Methods

### Synthesis and fluorescence labeling of peptides

Peptide synthesis was performed using an automated peptide synthesizer (Liberty, CEM, Kamp-Lintfort, Germany) or by linear solid-phase peptide synthesis (SPPS) on Fmoc-Tyr(^t^Bu)-Wang resin according to Amblard et al. [24]. Peptides with the amino acid sequence LGQQQPFPPQQPY and LQLQPFPQPQLPY are referred to as P31–43 and P56–68, respectively. The resulting product was purified by preparative HPLC on a Merck LiChrosorb RP-8 column (250×4.6 mm) with a MeCN gradient (5–95% over 15 min) in H_2_O (P31–43: flow rate = 3 mL/min, retention time = 3.0 min; P56–68: flow rate = 3 mL/min, retention time = 2.5 min). The purified product was analysed by LCMS (P31–43: flow rate = 0.2 mL/min; retention time = 15 min, P56–68: flow rate = 0.2 mL/min; retention time = 17 min). These peptides were subsequently labeled at their *N*-terminus with Promofluor–488 (PF–488) (PromoKine, Heidelberg, Germany). In a typical experiment, 15 mg (9.82 µmol) of the peptide P31–43 were dissolved in dry dimethyl sulfoxide (DMSO) under a nitrogen atmosphere. The pH was adjusted to 8 with triethylamine and the solution was cooled to 0°C. 6.1 mg (9.82 µmol) PF–488 *N-*hydroxysuccinimide (NHS) -ester in 8 mL dry DMSO were added and the mixture was stirred for 48 h at room temperature in the dark. The solvent was removed by freeze drying. The resulting crude product was purified by preparative HPLC (PF-P31–43: RP-18 column, flow rate = 2 mL/min, retention time = 7.1 min; PF-P56–68: RP-8 column, flow rate = 2 mL/min, retention time = 2.4 min) and identified by LCMS (PF-P31–43: flow rate = 0.2 mL/min; retention time = 17 min, PF-P56–68: flow rate = 0.2 mL/min; retention time = 19 min).

### Cell culture

Human intestinal epithelial cells (Caco–2) were obtained from ATCC (Manassas, VA, USA) and cultured at 37°C with 5% CO_2_ and 95% humidity in DMEM supplemented with 1% glutamine, 1% sodium pyruvate, 1% nonessential amino acids (NAA) and 10% heat inactivated fetal calf serum (FCS) (Invitrogen, Darmstadt, Germany). Culture media were changed every two to three days. Cells used for experiments were at passage 10–30.

**Figure 1:**
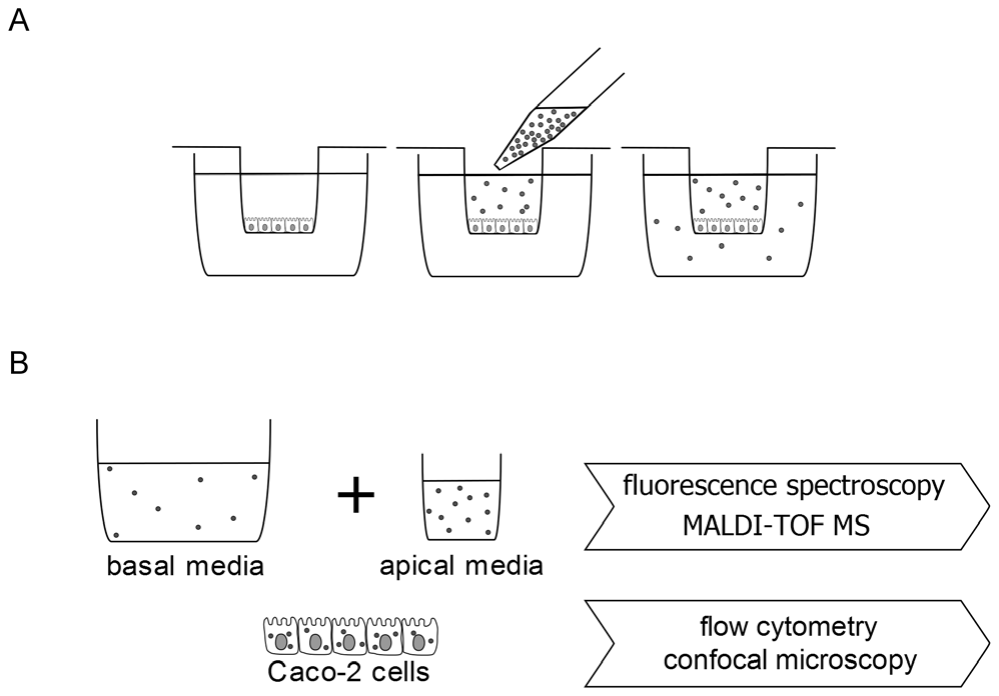
Experimental setup. (A) A differentiated Caco-2 Monolayer was incubated with fluorescence labeled P31–43 or P56–68 on the apical side of the cells. (B) After 3 h or 24 h incubation the apical and basal cell media were analyzed by fluorescence spectroscopy and MALDI-TOF-MS. The Caco-2 cells were analyzed by flow cytometry and confocal microscopy.

The experimental setup is illustrated in Figure 1. For transport experiments differentiated Caco-2 cells forming an intact monolayer were used. Therefore, cells were seeded at a density of 2.25×10^5^ cells/cm^2^ on cell culture filter inserts (ThinCert, culture surface: 33.6 mm^2^, pore size: 0.4 µm) in 24-well plates (Greiner Bio One, Frickenhausen, Germany) and cultured with supplemented DMEM including 1% penicillin streptomycin (Invitrogen) and 20% FCS until 14–17 days after they had reached confluence.

Before the start and at the end of the transport experiments, the integrity of the cell monolayer was tested by measuring transepithelial electric resistance (TEER) on a volt-ohm meter (Millicell ERS, Millipore, Schwalbach, Germany). Monolayers with insufficient TEER (<450 Ω/cm^2^) were omitted.

Cells were pre-incubated with transport medium (Hank's Balanced Salt Solution (HBSS) with Ca^2+^, Mg^2+^ and glucose) (Invitrogen) (200 µl in the apical, 900 µl in the basal compartment of the transwell system) for 30 min at 37°C. Methyl-β-cyclodextrin (MβCD; Sigma-Aldrich, Munich, Germany; 2 mmol/L) was added to the apical compartment to inhibit endocytosis. Then, PF–488 labeled GP dissolved in 100 µl transport medium were added on the apical side of the cells at a total concentration of 32 µmol/L or 145 µmol/L. Cells were incubated at 37°C for 3 or 24 h. Thereafter, media from both compartments (apical and basal) were removed to be further analyzed by fluorescence spectroscopy and MALDI–TOF–MS; cell inserts were prepared for flow cytometry and confocal laser scanning microscopy.

To inhibit transcellular antigen uptake, experiments were done at 4°C for 3 h. Caco–2 monolayer were pre-incubated with cold transport medium (200 µl apical, 900 µl basal) at 4°C for 10 min. As described above, PF–488 labeled GP (32 µmol/L) were added and the cells were incubated 3 h at 4°C. Then, cells were washed using ice cold PBS (Invitrogen). Since the monolayer integrity was disrupted after 3 h incubation at 4°C, the cell layer, but not culture media were further analyzed by using flow cytometry and confocal laser scanning microscopy.

### Fluorescence spectroscopy

After incubation of Caco–2 cells with PF–488 labeled GP, the apical and basal media were collected and measured by fluorescence spectroscopy (Fluoroskan Ascent FL, Thermo Scientific, Langenselbold, Germany). The fluorescence intensity of PF–488 was determined at a wavelength of 485 nm for excitation and 538 nm for emission. The measured fluorescence intensity was corrected by the background fluorescence of the incubation media. The translocation of PF–488 labeled peptides into the basal compartment was quantified in relation to the apical fluorescence intensity. To identify the corresponding peptides, MALDI-TOF-MS analysis was done.

### MALDI–TOF-MS

For mass spectrometric analyses peptides were concentrated and desalted using C18 ZipTips (Millipore). MALDI–TOF–MS analysis of the apical and basal cell culture media was performed using a Bruker Ultraflex I instrument (Bruker Daltonics, Bremen, Germany) with 2,5-dihydroxybenzoic acid (DHB)/methylenbisphosphonic acid as matrix. For calibration an external standard peptide mixture (Bruker Daltonics) was used. MS-spectra were processed by the software FlexAnalysis (version 3.0, Bruker Daltonics). The peptide sequences of P56–68 and P31–43 as well as fragments of these peptides were deduced from the detected molecular mass considering the H^+^, Na^+^ and K^+^ adducts. Masses were assigned to peptides allegeable to cleavage of GP. Detected GP were quantified by calculating the relative area under the curve (AUC). The values of three independent experiments are given as mean values. Based on the affinity of peptidases localised on the intestinal brush border membranes for certain amino acid linkages [16] the degradation pathways of P31–43 and P56–68 were reconstructed.

### Flow cytometry

After removal of the apical and basal media, Caco–2 cells were rinsed with PBS and dissociated with trypsin/EDTA solution (Invitrogen) supplemented with 1.9 mmol/L sodium EDTA (Sigma-Aldrich) for 8 min to get a single-cell suspension. Trypsin was inhibited by adding culture media and the cells were washed with PBS. After resuspension in PBS the cells were analyzed by flow cytometry (Guava easyCyte mini, Millipore, Hayward CA, USA) and the software CytoSoft 4.2.1 (Guavatechnologies, Hayward CA, USA). The arithmetic mean of fluorescence intensity determined for 5,000 cells was used to calculate the mean fluorescence intensity (MFI) of three independent experiments. The MFI of cells incubated without PF–488 labeled peptides was used to subtract background staining.

### Confocal laser scanning microscopy

Caco–2 cells were rinsed twice with PBS and fixed with 3% paraformaldehyde solution (Sigma-Aldrich) for 5 min. Nuclear staining was done by Hoechst 33342 solution (Invitrogen) for 5 min. Thereafter, cells were washed twice with PBS. To mount Caco-2 cells on a slide, the filter covered with the cell monolayer was cut out and mounted on a cover slip using Prolong Gold (Invitrogen) as mounting reagent. Confocal laser scanning microscopy was done on a Nikon Eclipse TE2000 equipped with EZ-C1 3.80 software (Nikon, Düsseldorf, Germany) with 100× magnification. Excitation was done by Argon (488 nm) and HeNe (543 nm) lasers. Three-dimensional pictures were generated using the software NIS-Elements 3.20 (Nikon).

### Statistics

Statistical analysis was done using the software GraphPad Prism 4.03 (San Diego, CA, USA). Each experiment was performed at least three times and expressed as means ± SD. To compare treatment groups, the two-tailed unpaired student's t-test was used. Statistical significance was defined at *p*<0.05.

## Results

### Translocation of GP

**Figure 2:**
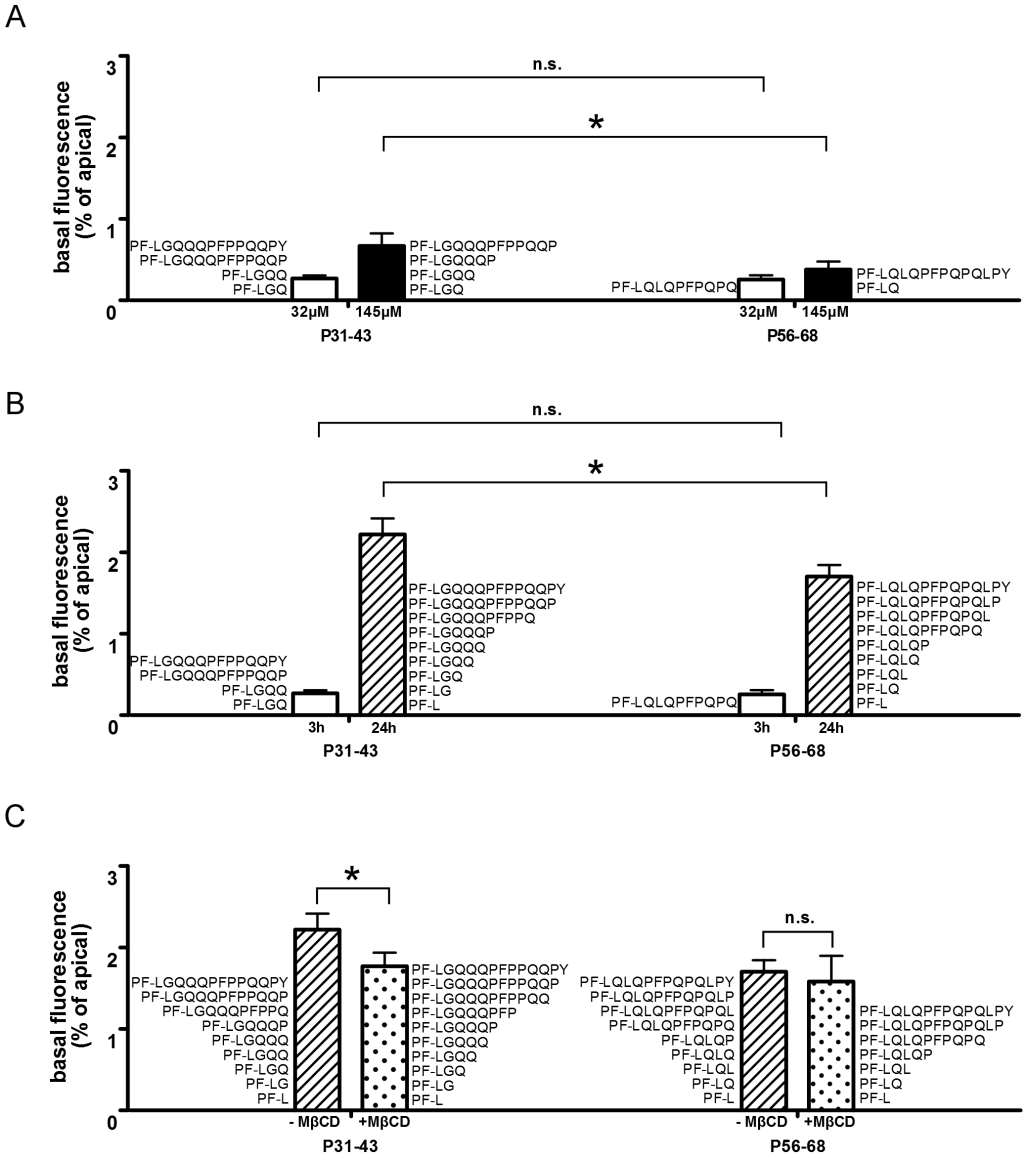
Epithelial translocation in Caco-2 monolayers of fluorescence labeled gliadin peptides measured by fluorescence spectroscopy and MALDI-TOF-MS. Fluorescence intensity and mass spectrometric identified peptides in the basal cell culture media (A) after incubation with fluorescence labeled P31–43 and P56–68 (32 µmol/L and 145 µmol/L) for 3 h, (B) after incubation with 32 µmol/L fluorescence labeled P31–43 and P56–68 for 3 h and 24 h, (C) after incubation with 32 µmol/L fluorescence labeled P31–43 and P56–68 with and without MβCD for 24 h. Basal fluorescence is given as % of apical fluorescence intensity. Results are shown as mean ± SD; *p<0.05; n.s.: not significant.

To examine whether or not small immunogenic and toxic GP are able to cross an intestinal epithelial barrier, a differentiated Caco–2 monolayer was incubated with PF-488 labeled P31–43 and P56–68 on the apical side of the cells. The translocation of the peptides was evaluated by fluorometric detection of PF-488 in the incubation media followed by identification of the peptides with and without fluorescent label by MALDI-TOF-MS. By measurement of TEER before and at the end of each transport experiment we observed that the integrity of the monolayer was not affected over the time of incubation. Fluorometric analyses of the cell media revealed significantly higher basal fluorescence intensities when cells were incubated for 3 h with 145 µmol/L P31–43 compared to cells incubated with an equimolar amount of P56–68 (0.7 and 0.4% of the apical fluorescence intensity, respectively, p<0.05). At a lower concentration of GP (32 µmol/L), fluorescence intensity in the basal media was lower but no differences were found between P31–43 and P56–68 (0.3% for both peptides) (Fig. 2A). However, after 24 h incubation at the same concentration significant differences were observed depending on the peptide used, i.e., fluorescence intensity in the basal compartment was significantly higher after incubation of cells with P31–43 compared to P56–68 (2.2 vs. 1.7%, respectively) (Fig. 2B). Using MβCD as an inhibitor of endocytosis, translocation was decreased in cells incubated with P31–43 by approximately 20%; no significant difference was observed with P56–68 (1.8 and 1.6%, respectively) (Fig. 2C). To verify that the detected fluorescence signals correspond to labeled GP, MALDI-TOF-MS analyses were applied. We found that intact peptides as well as fragments carry the fluorescence label (PromoFluor-488) in the apical and basal cell culture media (Fig. 2, Table S1–S4). Thus, it was shown that after 3 h of incubation, the majority of the fluorescence labeled peptides in the apical media consisted of intact P31–43 and P56–68. After 24 h of incubation, however, most fluorescence labeled peptides were degraded by cleavage of at least one amino acid. In the basal media showing fluorescence signals, trace amounts of intact P31–43 and P56–68 as well as fragmented peptides were identified by MALDI-TOF.

### Cellular uptake of GP

**Figure 3:**
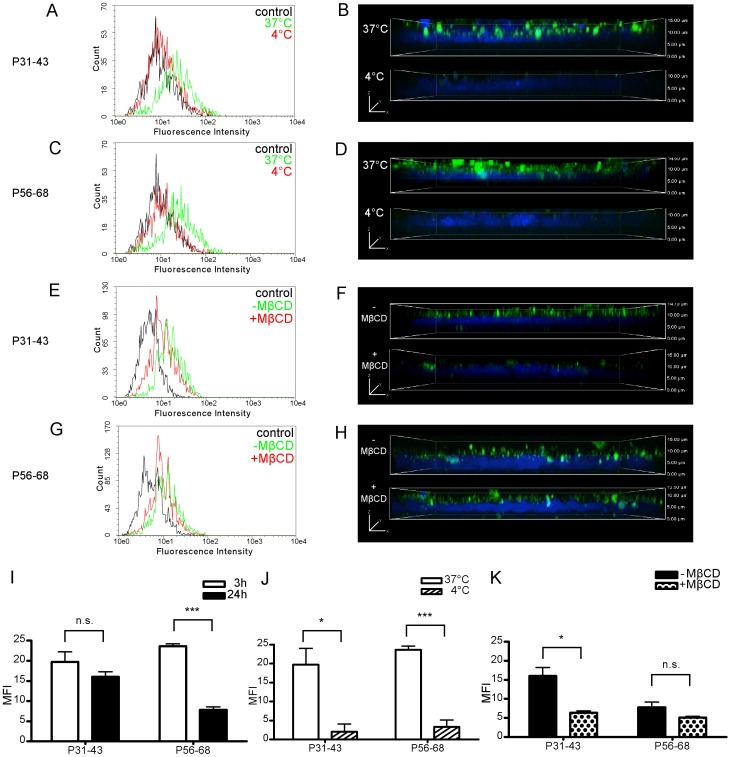
Cellular uptake of fluorescence signal in Caco–2 cells incubated with fluorescence labeled GP. Flow cyctometry (A+C) and confocal microscopy (B+D) of Caco–2 cells after 3 h incubation with P31–43 (A+B) and P56–68 (C+D) at 37°C and 4°C. Flow cyctometry (E+G) and confocal microscopy (F+H) of Caco–2 cells after 24 h incubation with P31–43 (E+F) and P56–68 (G+H) with (+MβCD) and without methyl-β-cyclodextrin (−MβCD). To detect autofluorescence of Caco–2 cells, non-treated cells were analysed and used as controls. Mean fluorescence intensity (MFI) of cells analysed by flow cytometry (I-K): Comparison of MFI of Caco–2 cells after 3 h and 24 h incubation with P31–43 and P56–68 (I). Comparison of MFI of Caco–2 cells after 3 h incubation with P31–43 and P56–68 at 37°C and 4°C (J). Comparison of MFI of Caco–2 cells after 24 h incubation with P31–43 and P56–68 with and without MβCD (K). Results are shown as mean ± SD; *p<0.05, ***p<0.005.

Flow cytometry and confocal microscopy revealed intracellular uptake of fluorescence labeled antigens for both GP tested. Cell associated MFI increased to a similar extent after 3 h incubation with P31–43 and P56–68 at a concentration of 32 µmol/L. Control experiments at 4°C showed decreased MFI for both peptides tested (Fig. 3A, C and J). Confocal microscopy was used to further examine the exact localization of the fluorescence signal within the cells. Only weak fluorescence signals were detected at 4°C. After incubation at 37°C, however, fluorescent vesicles were found at the apical side of the cells for both peptides (Fig.3B and D). Incubation of Caco-2 cells with P56–68 for 24 h led to decreased cell associated MFI compared to 3 h experiments. In contrast, no significant difference was observed for P31–43 with regard to incubation times (Fig. 3I). Flow cytometric analysis revealed a reduced MFI for cells incubated with P31–43 for 24 h in combination with MβCD, whereas no further decrease by MβCD was found with P56–68 (Fig. 3E-H, K).

### Digestion of P31–43 and P56–68 by Caco-2 cells

To further investigate the structural integrity of the peptides throughout the translocation process, MS analyses were performed.

**Figure 4:**
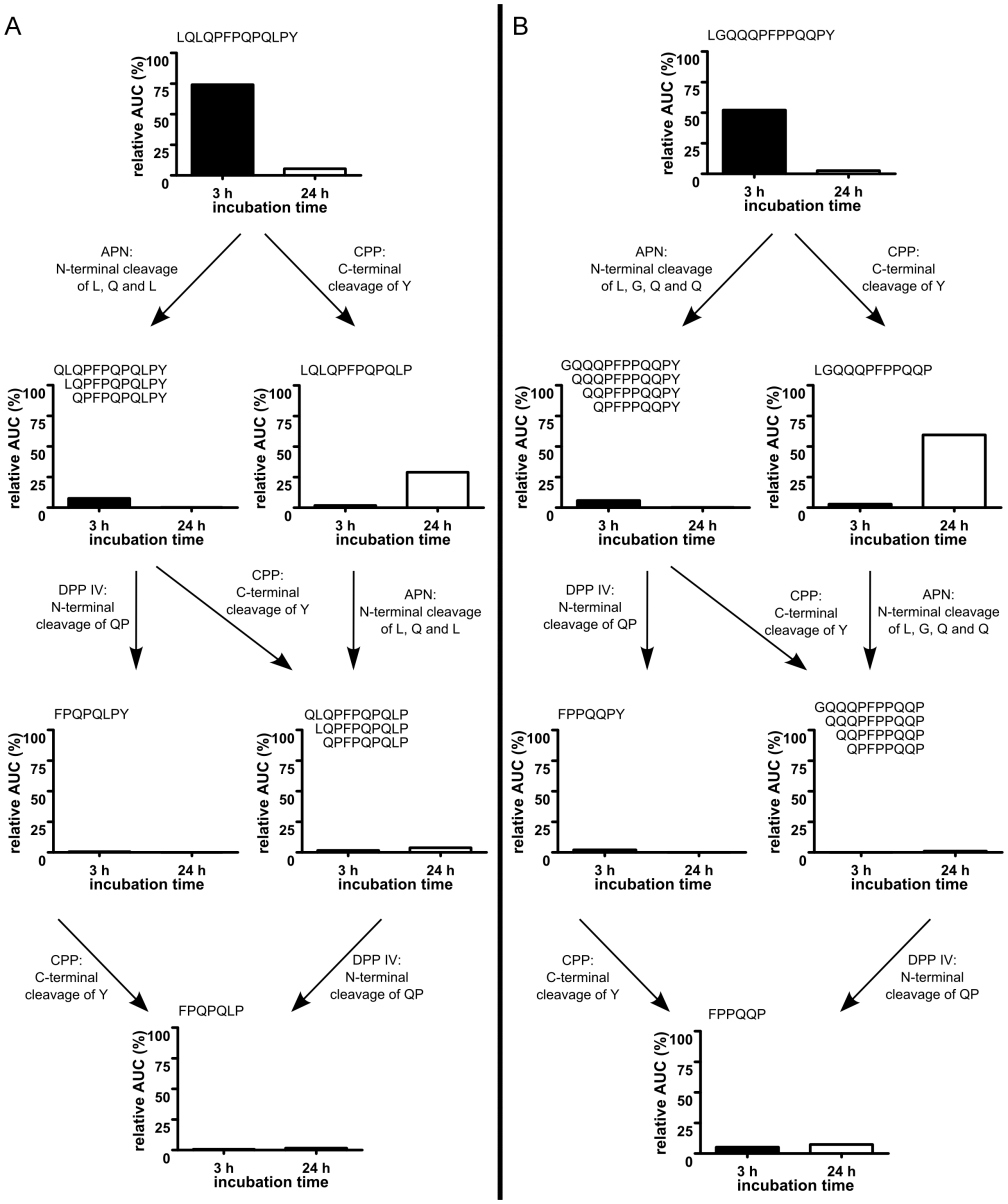
Digestion of P56–68 and P31–43 by Caco–2 cells. MALDI–TOF–MS analysis of digestion products of P56–68 (LQLQPFPQPQLPY) (A) and P31–43 (LGQQQPFPPQQPY) (B) on the apical side of Caco–2 monolayers. The degradation pathway was reconstructed considering the membrane peptidases APN, CPP and DPP IV.

Using MALDI-TOF-MS, we showed that fluorescence labeled GP were partly cleaved at the apical side of the intestinal cells. Based on the MS-profiles (Tab. S1–S4) and the known amino acid affinities of peptidases localized in the brush border membrane [25], [16], a degradation pathway of the peptides was reconstructed. MALDI–TOF–MS revealed that the majority of P31–43 (76%) and P56–68 (84%) remaining in the apical compartment were still intact after 3 h. However, after 24 h, most of the *C*-terminal tyrosine residues of the P31–43 (83%) and P56–68 (72%) were cleaved, presumably by carboxypeptidase P (CPP). After 3 h of incubation cleavage of *N*–terminal leucine, glutamine and leucine residues in P56–68 were detected. Sequences attributed to degradation by aminopeptidase N (APN) contributed only little to the identified peptide fragments after 24 h incubation time. In P31–43, even four *N*–terminal amino acids (leucine (L) glycine (G), glutamine (Q), glutamine (Q)) were hydrolysed, probably mediated by APN. Further fragments as potential cleavage products of dipeptidyl peptidase IV (DPP IV), CPP and APN were also detected in small amounts after 3 h and 24 h (Fig. 4).

There was no indication for significant translocation of the intact P56–68 or fragments of this peptide when the cell monolayer had been incubated with 32 µmol/L of the peptide for 3 h. However, after 24 h the intact peptide and also the fragment LQLQPFPQPQLP (P56–67), a potential cleavage product catalyzed by CPP, were found. Products derived from CPP-cleavage of P31–43 after incubation, e.g., LGQQQPFPPQQP (P31–42) were detected in the basal media after 3 and 24 h. In addition, smaller cleavage products as well as the intact GP were identified.

## Discussion

In celiac disease certain GP sequences seem to influence inflammatory processes. Toxic GP like P31–43 are thought to induce mucosal damage by activation of the innate immune response whereas immunogenic GP like P56–68 contain epitopes which were shown to induce an adaptive immune response by stimulation of DQ2 restricted T-cell clones derived from celiac patients [6], [26]. Until now it is not fully understood how toxic and immunogenic GP can pass the epithelial barrier into the *lamina propria* region to induce immune responses.

The aim of our study was to investigate the epithelial translocation of P31–43 and P56–68 which are assumed to be minimal motifs to induce innate immune responses or T-cell stimulation, respectively. Mamone et al. [27] reported that P56–68 was present in gastric-pancreatic digests of α-gliadin. They further observed that P31–55 which was also found after digestion of α-gliadin was able to pass the epithelial barrier in an intact form. Therefore, we hypothesized that P31–43 crosses the epithelial barrier in an intact form, since P31–43 was previously shown not to be transported into late endosomes or lysosomes [21]. In further studies using intestinal biopsies from control and celiac patients the immunogenic P57–68 was found to be completely degraded after mucosal to serosal transport. In contrast to biopsies of celiac patients, P57–68 was already partially degraded by brush-border peptidases in control subjects; the longer peptides P56–88 and P31–49 were not degraded by brush-border peptidases on the apical side but during the transport from the mucosal to the serosal side [19], [28].

However, the use of intestinal biopsies from celiac and control patients to investigate transport and degradation of GP comprises a complex interplay between various cell types and hardly allows to reveal the role the enterocytes in the metabolic fate of GP and thus their role in antigen transport and presentation.

To investigate the involvement of enterocytes experiments using Caco-2 cells as a model for the epithelial barrier were carried out. Schumann et al. [29] who also used this cell model observed epithelial transcytosis and partial degradation of P56–88, a longer form of P56–68. In addition, Iacomino et al. [30] showed that the toxic P31–55 which corresponds in its first 13 amino acids to P31–43 which we used in our experiments was resistant against degradation and able to pass an intact Caco–2 monolayer.

In our experiments, information given by fluorescence spectroscopy, flow cytometry and confocal microscopy suggested an intracellular as well as a transcellular uptake of fluorescence labeled peptides. However, MALDI-TOF-MS revealed a cleavage of P31–43 and P56–68 already at the apical side of the cells as a function of time. Thus, tracking of a fluorescence label does not necessarily correlate with a transport of intact antigens across the intestinal cell barrier but can also represent fluorescence labeled fragments of the peptide.

We were able to confirm the results of Barone et al. [31] and Lübbing et al. [21] demonstrating intracellular uptake of fluorescence labeled P31–43 and P56–68 in Caco–2 cells. Furthermore, we found that, as opposed to P31–43, incubation with P56–68 for longer time periods or with increasing concentration resulted in a saturation of uptake or translocation indicating the ability of P31–43 or its fragments to overcome the epithelial barrier more easily (Fig. 2A, B, 3J).

To investigate the transport mechanism for GP, MβCD was used as an inhibitor of endocytosis. MβCD is known for its cholesterol extracting properties thereby inhibiting clathrin-independent endocytosis, but also an inhibitory effect on clathrin-mediated endocytosis is discussed [32]–[34]. In our experiments, MβCD down-regulated the uptake and translocation of peptides in cells incubated with P31–43 but not with P56–68, suggesting a clathrin-independent endocytotic uptake of P31–43 or its fragments (Fig. 2C, 3). This is in agreement with Caputo et al. [35] who observed a reduced endocytosis of fluorescence signals in cells incubated with labeled P31–43 but only a slightly reduced endocytosis with labeled P57–68. Schumann et al. [29], however, reported the inhibition of translocation of intact fluorescence labeled P56–88 by using MβCD.

Similar to different notions regarding the intracellular uptake of GP there are also controversial data about the endocytotic transport of P31–43 and P56–68. Previous studies using fluorescence labeled peptides or immunostaining indicated that P56–68 was transported into late endosomes whereas P31–43 remained in early endosomes [20], [21], [36]. However, only using the tracking of fluorescence signals may limit the conclusions that can be drawn with regard to the structural integrity of the peptides. Thus, we also performed MALDI-TOF-MS analyses revealing time dependent cleavage of both GP on the apical side of the cells. As has previously been shown by Hausch et al. [16], immunogenic GP can be partly digested by brush-border membrane peptidases of rat small intestinal mucosa. Their intact P57–68 had been degraded with a half-life of 35 min but degraded peptides with a high stability of the PFPQPQLP motif were observed over prolonged incubation times. Enzymes most likely involved in the cleavage are APN, CPP and DPP IV [16]. Our MS results revealed that the majority of P56–68 and P31–43 exposed to intestinal cells remained intact over 3 h. However, after 24 h, C-terminal tyrosine was cleaved in the majority of both peptides suggesting a degradation catalyzed by CPP (Fig. 4).

Considering the translocation to the basal side of the cells after 3 h incubation neither intact P56–68 nor fragments originating from degradation by APN, CPP or DPP IV were identified.

Using 24 h incubation, little intact P56–68 and mostly P56–67 was detected in the basal compartment. Arentz-Hansen et al. [37] supposed that the C-terminal tyrosine of P57–68 is critical for binding to HLA-DQ2 and cleavage of this amino acid abolished the binding to HLA-DQ2 molecules and thus the recognition by T-cells. They have shown, that stepwise truncation of this peptide at the N-terminal position also let to a successive loss of HLA-DQ2 binding affinity and recognition by T-cells. Therefore, we suppose that P56–67 would not be able to induce inflammatory responses in celiac patients.

Toxic GP such as P31–43 are thought to induce not only the production of IL–15 in the *Lamina propria* leading to the activation of intraepithelial lymphocytes and the destruction of enterocytes [12] but also to stimulate the secretion of inflammatory cytokines in monocytes; thus, having an effect on the innate immune response in leukocytes [38]. When we incubated immature dendritic cells with GP, we observed a similar, i.e. pro-inflammatory effect for P31–43 but not for P56–68 (Fig. S1). However, applying basal cell culture media of Caco–2 cells incubated with P31–43 did not significantly increase cytokine production (Fig. S2). This might be an indication that the amount of intact P31–43 and its fragments in the basal compartment was too low to stimulate cytokine secretion

In addition to transcellular pathways, paracellular transport mechanisms may be involved in the process of translocation of GP across the epithelial barrier. It was shown that gliadin enhanced paracellular permeability [39]–[41]. Clemente et al. [42] reported that the toxic P31–55 but not a non-toxic GP increased the paracellular leak in rabbit intestinal mucosa. In contrast, experiments with celiac biopsies indicated that there was no major paracellular flux of the toxic P31–49 and the immunogenic P56–88 [28].

In contrast to the existing hypothesis that toxic GP stay intact while passing the epithelial barrier we have shown for the first time that, apart from the immunogenic P56–68, the toxic P31–43 can be partly degraded by enterocytes as well. According to the structural analyses by MALDI-TOF-MS, however, only small amounts of intact toxic and immunogenic peptides were able to pass the Caco–2 monolayer.

So far, previous investigations concentrated on various peptides with a different length which were all bigger than the ones we investigated. Therefore, the data are difficult to compare. From those previous studies the major conclusions were that larger peptides can be digested and that it might be better to use smaller and non-digestible peptides for further pre-clinical and clinical studies. However, from our experiments it is obvious that even smaller peptides can be digested prior to the translocation across the epithelial barrier.

To conclude, (i) we report for the first time on the translocation and degradation of an immunogenic as well as of a toxic GP with the same length; (ii) we found a higher translocation of P31–43 or its fragments compared with P56–68; (iii) in addition, we also observed for the first time the degradation of the toxic GP P31–43 at the apical side of epithelial cells; and (iv) we measured an increased secretion of IL-8 und TNF-α by dendritic cells incubated with intact P31–43; (iv) in contrast, no effect was observed by either P56–68 or the peptides which had passed the intestinal cell layer. Thus, the chain length of GP as well as the integrity of the epithelial barrier seem to be critical for an increased uptake of GP and the subsequent inflammatory immune response.

## Supporting Information

Figure S1 **Effect of P31–43 and P56–68 on cytokine secretion (IL-8, TNF-α) by immature dendritic cells (iDC).** iDC were generated from CD14+ PBMCs by a 5-day incubation with 500 U/mL IL-4 and 1000 U/mL GM-CSF in RPMI media with 10% human serum. Then, iDC were incubated with 100 µg/mL P31–43 or P56–68 in RPMI+2% human serum for 24 h. IL-8 (A) and TNF-α (B) secretion in the media were analyzed by a commercially available sandwich ELISA (R&D, Wiesbaden, Germany). Each experiment was performed three times; data are given as mean ± SD; only significant differences are shown as *p<0.05, **p<0.01.
(TIFF)

Figure S2 **Effect of the basal media - after incubation of a Caco-2 monolayer with P31–43 and P56–68 - on cytokine secretion (IL-8, TNF-α) by immature dendritic cells (iDC).** iDC were generated from CD14+ PBMCs by 5-day incubation with 500 U/mL IL-4 and 1000 U/mL GM-CSF in RPMI media with 10% human serum. iDC were incubated 24 h with basal media of Caco-2 monolayers after they had been exposed to PF-488 labeled P31–43 and P56–68 on the apical side of the cells. IL-8 (A) and TNF-α (B) secretion in the media was analyzed by sandwich ELISA. Each experiment was performed three times; data are given as mean ± SD.
(TIFF)

Table S1 **Fragments of P56–68 after 3 h incubation summarized in** **Figure 4** **A.** After incubation of the fluorescence labeled (PromoFluor-488, PF) P56–68 (PF-P56–68) at the apical side of the Caco-2 monolayer, P56–68 was partially cleaved into several fragments (A). Analysis of the basal media revealed translocation of few P56–68 fragments (B). Analysis of the molecular masses was done by MALDI-TOF-MS. Detected molecular masses were assigned to the masses of P56–68 and fragments thereof. The experiment was repeated 3 times (sample 1, 2 and 3).
(PDF)

Table S2 **Fragments of P56–68 after 24 h incubation summarized in** **Figure 4** **A.** After incubation of the fluorescence labeled (PromoFluor-488, PF) P56–68 (PF-P56–68) at the apical side of the Caco-2 monolayer, the majority of P56–68 was cleaved into several fragments (A). Analysis of the basal media revealed translocation of some intact P56–68 as well as fragments (B). Analysis of the molecular masses was done by MALDI-TOF-MS. The detected molecular masses were assigned to the masses of P56–68 and fragments thereof. The experiment was repeated 3 times (sample 1, 2 and 3).
(PDF)

Table S3 **Fragments of P31–43 after 3 h incubation summarized in** **Figure 4** **B.** After incubation of the fluorescence labeled (PromoFluor-488, PF) P31–43 (PF-P31–43) at the apical side of the Caco-2 monolayer, P31–43 was partially cleaved into several fragments (A). Analysis of the basal media revealed translocation of some intact P31–43 as well as fragments (B). Analysis of the molecular masses was done by MALDI-TOF-MS. The detected molecular masses were assigned to the masses of P31-43 and fragments thereof. The experiment was repeated 3 times (sample 1, 2 and 3).
(PDF)

Table S4 **Fragments of P31–43 after 24 h incubation summarized in** **Figure 4** **B.** After incubation of the fluorescence labeled (PromoFluor-488, PF) P31–43 (PF-P31–43) at the apical side of the Caco-2 monolayer, the majority of P31–43 was cleaved into several fragments (A). Analysis of the basal media revealed translocation of some intact P31–43 as well as fragments (B). Analysis of the molecular masses was done by MALDI-TOF-MS. The detected molecular masses were assigned to the masses of P31-43 and fragments thereof. The experiment was repeated 3 times (sample 1, 2 and 3).
(PDF)

## References

[1] Tjon JM, van Bergen J, Koning F. Celiac disease: how complicated can it get. Immunogenetics. 2010;62(10):641–651. 20661732 10.1007/s00251-010-0465-9 PMC2944025

[2] Schuppan D, Junker Y, Barisani D. Celiac disease: from pathogenesis to novel therapies. Gastroenterology. 2009;137(6):1912–1933. 19766641 10.1053/j.gastro.2009.09.008

[3] Jabri B, Sollid LM. Tissue-mediated control of immunopathology in coeliac disease. Nat. Rev. Immunol. 2009;9(12):858–870. 19935805 10.1038/nri2670

[4] Wieser H. Chemistry of gluten proteins. Food Microbiol. 2007;24(2):115–119. 17008153 10.1016/j.fm.2006.07.004

[5] Townley RR, Cornell HJ, Bhathal PS, Mitchell JD. Toxicity of wheat gliadin fractions in coeliac disease. Lancet. 1973;1(7816):1363–1364. 4122746 10.1016/s0140-6736(73)91679-6

[6] Ciccocioppo R, Di Sabatino A, Corazza GR. The immune recognition of gluten in coeliac disease. Clin Exp Immunol. 2005;140(3):408–416. 15932501 10.1111/j.1365-2249.2005.02783.x PMC1809391

[7] van de Wal, Y, Kooy YM, van Veelen, P A, Peña SA, Mearin LM, et al Small intestinal T cells of celiac disease patients recognize a natural pepsin fragment of gliadin. Proc. Natl. Acad. Sci. U.S.A. 1998;95(17):10050–10054. 9707598 10.1073/pnas.95.17.10050 PMC21459

[8] van de Wal, Y, Kooy YM, van Veelen P, Vader W, August SA, et al Glutenin is involved in the gluten-driven mucosal T cell response. Eur. J. Immunol. 1999;29(10):3133–3139. 10540324 10.1002/(SICI)1521-4141(199910)29:10<3133::AID-IMMU3133>3.0.CO;2-G

[9] Sjöström H, Lundin KE, Molberg O, Körner R, McAdam SN, et al Identification of a gliadin T-cell epitope in coeliac disease: general importance of gliadin deamidation for intestinal T-cell recognition. Scand. J. Immunol. 1998;48(2):111–115. 9716100 10.1046/j.1365-3083.1998.00397.x

[10] van de Wal, Y, Kooy Y, van Veelen P, Peña S, Mearin L, et al Selective deamidation by tissue transglutaminase strongly enhances gliadin-specific T cell reactivity. J. Immunol. 1998;161(4):1585–1588. 9712018

[11] Vader W, Kooy Y, van Veelen P, Ru A de, Harris D, et al The gluten response in children with celiac disease is directed toward multiple gliadin and glutenin peptides. Gastroenterology. 2002;122(7):1729–1737. 12055577 10.1053/gast.2002.33606

[12] Maiuri L, Ciacci C, Ricciardelli I, Vacca L, Raia V, et al Association between innate response to gliadin and activation of pathogenic T cells in coeliac disease. Lancet. 2003;362(9377):30–37. 12853196 10.1016/S0140-6736(03)13803-2

[13] Sturgess R, Day P, Ellis HJ, Lundin KE, Gjertsen HA, et al Wheat peptide challenge in coeliac disease. Lancet. 1994;343(8900):758–761. 7907731 10.1016/s0140-6736(94)91837-6

[14] Marsh MN, Morgan S, Ensari A, Wardle T, Lobley R, et al In vivo activity of peptides 31–43, 44–55, 56–68 of a-gliadin in gluten sensitive enteropathy(GSE). Gastroenterology. 1995;108(4):A871.

[15] Silk DB, Grimble GK, Rees RG. Protein digestion and amino acid and peptide absorption. Proc Nutr Soc. 1985;44(1):63–72. 3885229 10.1079/pns19850011

[16] Hausch F, Shan L, Santiago NA, Gray GM, Khosla C. Intestinal digestive resistance of immunodominant gliadin peptides. Am J Physiol Gastrointest Liver Physiol. 2002;283(4):G996–G1003. 12223360 10.1152/ajpgi.00136.2002

[17] Pappenheimer JR, Dahl CE, Karnovsky ML, Maggio JE. Intestinal absorption and excretion of octapeptides composed of D amino acids. Proc Natl Acad Sci U S A. 1994;91(5):1942–1945. 8127911 10.1073/pnas.91.5.1942 PMC43280

[18] Snoeck V, Goddeeris B, Cox E. The role of enterocytes in the intestinal barrier function and antigen uptake. Microbes Infect. 2005;7(7–8):997–1004. 15925533 10.1016/j.micinf.2005.04.003

[19] Matysiak-Budnik T, Candalh C, Dugave C, Namane A, Cellier C, et al Alterations of the intestinal transport and processing of gliadin peptides in celiac disease. Gastroenterology. 2003;125(3):696–707. 12949716 10.1016/s0016-5085(03)01049-7

[20] Zimmer K, Fischer I, Mothes T, Weissen-Plenz G, Schmitz M, et al Endocytotic segregation of gliadin peptide 31–49 in enterocytes. Gut. 2010;59(3):300–310. 19654123 10.1136/gut.2008.169656

[21] Lubbing N, Barone MV, Rudloff S, Troncone R, Auricchio S, et al Correction of gliadin transport within enterocytes through celiac disease serum. Pediatr Res. 2011;70(4):357–362. 21705964 10.1203/PDR.0b013e31822a31e7

[22] Wilson G, Hassan IF, Dix CJ, Williamson I, Shah R, et al Transport and permeability properties of human Caco-2 cells. An in vitro model of the intestinal epithelial cell barrier. J Control Release. 1990;11(1–3):25–40.

[23] Rousset M. The human colon carcinoma cell lines HT-29 and Caco-2. two in vitro models for the study of intestinal differentiation. Biochimie. 1986;68(9):1035–1040. 3096381 10.1016/s0300-9084(86)80177-8

[24] Amblard M, Fehrentz JA, Martinez J, Subra G. Methods and protocols of modern solid phase Peptide synthesis. Mol Biotechnol. 2006;33(3):239–254. 16946453 10.1385/MB:33:3:239

[25] Bai JP. Distribution of brush-border membrane peptidases along the rat intestine. Pharm. Res. 1994;11(6):897–900. 7937532 10.1023/a:1018946228432

[26] Londei M, Maiuri L. Gliadin as stimulator adaptive and innate immune responses in celiac disease. J. Pediatr. Gastroenterol. Nutr. 2004;39 Suppl 3:S729. 15167363 10.1097/00005176-200406003-00006

[27] Mamone G, Ferranti P, Rossi M, Roepstorff P, Fierro O, et al Identification of a peptide from alpha-gliadin resistant to digestive enzymes. implications for celiac disease. J Chromatogr B Analyt Technol Biomed Life Sci. 2007;855(2):236–241. 10.1016/j.jchromb.2007.05.009 17544966

[28] Menard S, Lebreton C, Schumann M, Matysiak-Budnik T, Dugave C, et al Paracellular versus transcellular intestinal permeability to gliadin peptides in active celiac disease. Am J Pathol. 2012;180(2):608–615. 22119716 10.1016/j.ajpath.2011.10.019

[29] Schumann M, Richter JF, Wedell I, Moos V, Zimmermann-Kordmann M, et al Mechanisms of epithelial translocation of the alpha(2)-gliadin-33mer in coeliac sprue. Gut. 2008;57(6):747–754. 18305066 10.1136/gut.2007.136366

[30] Iacomino G, Fierro O, D′Auria S, Picariello G, Ferranti P, et al Structural analysis and Caco-2 cell permeability of the celiac-toxic A-gliadin peptide 31–55. J Agric Food Chem. 2013;61(5):1088–1096. 23298305 10.1021/jf3045523

[31] Barone MV, Nanayakkara M, Paolella G, Maglio M, Vitale V, et al Gliadin peptide P31–43 localises to endocytic vesicles and interferes with their maturation. PLoS One. 2010;5(8):e12246. 20805894 10.1371/journal.pone.0012246 PMC2923621

[32] Rodal SK, Skretting G, Garred O, Vilhardt F, van Deurs B, et al Extraction of cholesterol with methyl-beta-cyclodextrin perturbs formation of clathrin-coated endocytic vesicles. Mol Biol Cell. 1999;10(4):961–974. 10198050 10.1091/mbc.10.4.961 PMC25220

[33] Weangsripanaval T, Murota K, Murakami Y, Kominami M, Kusudo T, et al Sodium cromoglycate inhibits absorption of the major soybean allergen, Gly m Bd 30K, in mice and human intestinal Caco-2 cells. J Nutr. 2006;136(11):2874–2880. 17056816 10.1093/jn/136.11.2874

[34] Vercauteren D, Vandenbroucke RE, Jones AT, Rejman J, Demeester J, et al The use of inhibitors to study endocytic pathways of gene carriers. optimization and pitfalls. Mol Ther. 2010;18(3):561–569. 20010917 10.1038/mt.2009.281 PMC2839427

[35] Caputo I, Barone MV, Lepretti M, Martucciello S, Nista I, et al Celiac anti-tissue transglutaminase antibodies interfere with the uptake of alpha gliadin peptide 31–43 but not of peptide 57–68 by epithelial cells. Biochim Biophys Acta. 2010;1802(9):717–727. 20553859 10.1016/j.bbadis.2010.05.010

[36] Barone MV, Zanzi D, Maglio M, Nanayakkara M, Santagata S, et al Gliadin-mediated proliferation and innate immune activation in celiac disease are due to alterations in vesicular trafficking. PLoS One. 2011;6(2):e17039. 21364874 10.1371/journal.pone.0017039 PMC3045409

[37] Arentz-Hansen H, Körner R, Molberg O, Quarsten H, Vader W, et al The intestinal T cell response to alpha-gliadin in adult celiac disease is focused on a single deamidated glutamine targeted by tissue transglutaminase. J Exp Med. 2000;191(4):603–612. 10684852 10.1084/jem.191.4.603 PMC2195837

[38] Jelínková L, Tucková L, Cinová J, Flegelová Z, Tlaskalová-Hogenová H. Gliadin stimulates human monocytes to production of IL-8 and TNF-alpha through a mechanism involving NF-kappaB. FEBS Lett. 2004;571(1–3):81–85. 15280021 10.1016/j.febslet.2004.06.057

[39] Sander GR, Cummins AG, Henshall T, Powell BC. Rapid disruption of intestinal barrier function by gliadin involves altered expression of apical junctional proteins. FEBS Lett. 2005;579(21):4851–4855. 16099460 10.1016/j.febslet.2005.07.066

[40] Lammers KM, Lu R, Brownley J, Lu B, Gerard C, et al Gliadin induces an increase in intestinal permeability and zonulin release by binding to the chemokine receptor CXCR3. Gastroenterology. 2008;135(1):194–204 e3. 18485912 10.1053/j.gastro.2008.03.023 PMC2653457

[41] Drago S, El Asmar R, Di Pierro M, Grazia Clemente M, Tripathi A, et al Gliadin, zonulin and gut permeability. Effects on celiac and non-celiac intestinal mucosa and intestinal cell lines. Scand J Gastroenterol. 2006;41(4):408–419. 16635908 10.1080/00365520500235334

[42] Clemente MG, Virgiliis S de, Kang JS, Macatagney R, Musu MP, et al Early effects of gliadin on enterocyte intracellular signalling involved in intestinal barrier function. Gut. 2003;52(2):218–223. 12524403 10.1136/gut.52.2.218 PMC1774976

