# Supplementary figures and images for: Epithelial Transport of Immunogenic and Toxic Gliadin Peptides In Vitro
Source: PLoS One. 2014 Nov 21;9(11):e113932. doi: 10.1371/journal.pone.0113932 (PMC4240668; doi:10.1371/journal.pone.0113932)

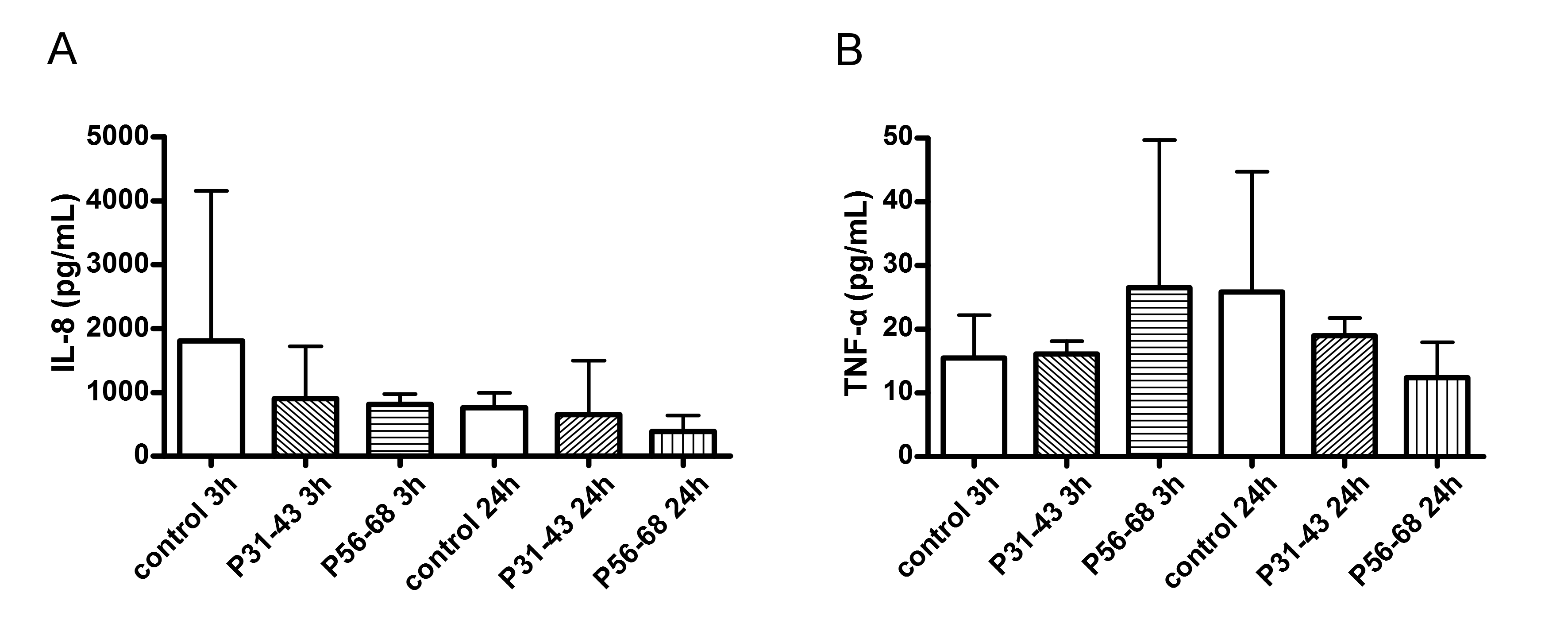

Supplement: Figure S2 — Effect of the basal media - after incubation of a Caco-2 monolayer with P31–43 and P56–68 - on cytokine secretion (IL-8, TNF-α) by immature dendritic cells (iDC). iDC were generated from CD14+ PBMCs by 5-day incubation with 500 U/mL IL-4 and 1000 U/mL GM-CSF in RPMI media with 10% human serum. iDC were incubated 24 h with basal media of Caco-2 monolayers after they had been exposed to PF-488 labeled P31–43 and P56–68 on the apical side of the cells. IL-8 (A) and TNF-α (B) secretion in the media was analyzed by sandwich ELISA. Each experiment was performed three times; data are given as mean ± SD. (TIFF) [file pone.0113932.s002.tiff]
