# Supplementary material for: Epithelial Transport of Immunogenic and Toxic Gliadin Peptides In Vitro
Source: PLoS One. 2014 Nov 21;9(11):e113932. doi: 10.1371/journal.pone.0113932 (PMC4240668; doi:10.1371/journal.pone.0113932)
Supplement: Table S1 — Fragments of P56–68 after 3 h incubation summarized in Figure 4 A. After incubation of the fluorescence labeled (PromoFluor-488, PF) P56–68 (PF-P56–68) at the apical side of the Caco-2 monolayer, P56–68 was partially cleaved into several fragments (A). Analysis of the basal media revealed translocation of few P56–68 fragments (B). Analysis of the molecular masses was done by MALDI-TOF-MS. Detected molecular masses were assigned to the masses of P56–68 and fragments thereof. The experiment was repeated 3 times (sample 1, 2 and 3). (PDF) [file pone.0113932.s003.pdf]

3h incubation of Caco-2 cells with PF-p56-68

A

apical sample 1

m/z	area	
517.0059276	599.9568891	
521.944871	292.883775	
546.1789847	1484.580529	
547.3072131	145.0385638	
548.2021578	145.1761304	
552.4134855	1041.11138	
557.9668111	539.9356505	
559.9410297	1354.159424	
561.8898935	147.3172663	
565.8574559	198.9619485	
573.7164149	70.57574546	
575.57596	113.250834	
579.9257707	825.9006793	
582.6524528	74.63483574	
602.4632645	35.5604411	
606.4922934	61.90928316	
607.7238252	192.7126787	
617.4571079	501.6042712	PQLPY
618.462551	762.5616548	
631.3712889	105.9283515	
633.3354737	37.46672836	
643.3336674	39.82563138	
652.2601917	72.85024888	
653.3426796	44.75677412	
659.3388028	113.3798988	
661.3576229	37.96576604	
675.3926908	167.5429093	
679.3981508	365.3799679	
681.3477057	70.9125755	
697.3979262	229.2284078	
713.3747669	61.16967595	
721.355226	81.04133562	
728.3718875	165.9887754	
731.3011914	583.4992174	PF-LQ
745.3911133	150.9338738	LQLQPF / QPQLPY
750.3956935	49.77310502	
760.5672151	34.72403515	
768.3565992	66.57330609	LQLQPF + Na / QPQLPY + Na
775.3610919	38.95872566	
777.450005	50.84653726	
790.4149055	758.9246204	
812.4402202	439.3738903	
826.4513369	75.54522459	LQPFPQP / PFPQPQL / FPQPQLP
844.3839384	360.2543133	PF-LQL
850.4501199	50.42946646	
868.3835369	43.36661509	PF-LQL + Na
876.4362289	23.08185372	
884.4180986	86.39818045	
890.4268692	29.64848759	
916.3865154	92.99728009	
923.5047502	112.4109922	PFPQPQLP
940.5240231	42.05752979	
953.4699162	335.4098596	
962.6860413	49.21111113	
965.4741539	110.9165699	
972.4479432	103.6013858	PF-LQLQ
975.5014906	327.0827494	
987.5106489	107.6690508	
989.517776	122.5191906	FPQPQLPY
991.4094587	71.64380173	
1036.558481	29.23090437	
1051.559994	159.6051061	QPFPQPQLP
1067.563021	49.65771232	LQLQPFPQP
1086.571135	263.044705	PFPQPQLPY
1128.523977	58.90863016	
1150.563601	99.61563688	
1164.631507	73.45865237	LQPFPQPQLP
1195.626145	36.24593379	LQLQPFPQPQ
1197.589049	20.3866782	
1199.598856	37.82477457	
1214.620547	771.0129874	QPFPQPQLPY
1264.574378	71.75823908	
1292.695426	126.1428136	QLQPFPQPQLP
1327.702223	116.0592101	LQPFPQPQLPY
1405.783934	96.01205764	LQLQPFPQPQLP
1455.769387	480.8042437	QLQPFPQPQLPY
1527.771446	48.09213786	
1553.755696	43.77397189	
1568.85184	534.9485162	LQLQPFPQPQLPY
1590.851828	32.26279126	LQLQPFPQPQLPY + Na
1606.711704	21.7340886	
1665.739001	37.95119026	
1735.756493	109.0485677	
1876.903665	68.46724066	PF-LQLQPFPQPQLP
1898.921006	28.15077278	PF-LQLQPFPQPQLP + Na
1998.926555	17.44772921	
2039.998145	1679.078566	PF-LQLQPFPQPQLPY
2042.094144	202.6456484	PF-LQLQPFPQPQLPY
2062.027131	392.6841891	PF-LQLQPFPQPQLPY + Na
2063.032985	557.1979976	PF-LQLQPFPQPQLPY (open) + Na
2092.093114	15.9630118	
2174.038281	23.82190642	

apical sample 2

m/z	area	
516.9962314	150.698064	
522.0158021	181.4678656	
546.2140786	175.0317659	
547.2356479	82.03487737	
552.4208637	237.8176547	
558.0020258	75.19085529	
559.9321561	178.9779143	
562.0078239	75.44627348	
565.9251739	53.91223774	
575.568745	57.95444743	
579.9548388	1157.750652	
597.6244358	23.16255806	
607.7635603	915.8011702	
617.4990349	167.858593	PQLPY
618.5048777	175.0573399	
631.39444	33.15872458	
659.3550794	38.87833903	
675.4189152	41.22902743	
679.4276413	62.58915291	
724.5216522	100.2756596	
728.386432	45.93916624	
731.3387716	74.04000154	PF-LQ
745.3903643	27.77920211	LQLQPF / QPQLPY
750.4074923	16.63709952	
768.3991436	34.50240523	LQLQPF + Na / QPQLPY + Na
777.4942064	44.24672503	
781.5593065	1366.592958	
790.4375418	102.7384465	
805.5323402	29.00943694	
809.5732072	44.12387935	
812.4480162	55.97872673	
825.5706394	57.27706217	LQPFPQP / PFPQPQL / FPQPQLP
844.3848269	59.50224963	PF-LQL
953.467941	52.73187177	
954.4139212	32.3444107	LQPFPQPQ / QPFPQPQL
975.4936993	67.29922869	
1086.562905	192.857486	PFPQPQLPY
1150.554093	17.0146002	
1214.609642	76.52527113	QPFPQPQLPY
1327.696822	16.82770526	LQPFPQPQLPY
1341.922536	62.38897683	
1455.749588	52.65744564	QLQPFPQPQLPY
1568.812924	127.1594195	LQLQPFPQPQLPY
1876.875221	43.50757533	PF-LQLQPFPQPQLP
1899.886019	24.166068	PF-LQLQPFPQPQLP + Na
2039.996384	3121.317475	PF-LQLQPFPQPQLPY
2062.03092	857.1588714	PF-LQLQPFPQPQLPY + Na
2063.031802	1150.63916	PF-LQLQPFPQPQLPY (open) + Na
2079.058255	20.70042545	PF-LQLQPFPQPQLPY + K
2175.056552	42.19000825	
2197.078668	29.82304232	

apical sample 3

m/z	area	
516.9601665	166.0942909	
521.9544308	86.50469614	
534.2044631	50.61293994	
538.4246271	62.54658721	
546.1619561	105.5191071	
557.9794022	44.32961921	
559.9611049	98.23816749	
565.8374723	36.18704829	
573.2723493	36.90686578	
575.5406374	260.9393938	
580.6200395	41.79225755	
582.6650767	52.01150891	
587.6405189	51.804068	
595.068382	27.19857108	
597.3761078	89.2026613	LQLQP
602.4918801	66.03709307	
617.4669823	125.6892099	PQLPY
618.4685842	116.6445257	
631.3753694	20.63649709	
640.674005	23.60203438	
653.3716397	53.04826872	
659.3437249	13.00016806	
665.3402873	18.51147261	
668.6643937	63.65781748	
675.4117781	22.28051409	
679.4092221	63.14208788	
681.3585336	57.36170664	
697.327495	30.53416957	
703.5854503	16.30690777	
713.3685251	19.85968203	
715.3952475	17.85884777	
727.3637887	19.7517605	
728.3795728	24.41762109	
731.3160515	71.89108781	PF-LQ
739.3618322	36.50100157	
743.3961062	47.95790135	
745.3986673	39.05547582	LQLQPF / QPQLPY
750.4074246	74.28550948	
753.3289418	18.70355521	
757.3621746	29.01319271	
768.3757552	566.9785253	
775.3850437	28.76231474	
777.4170473	30.72563322	
790.4294489	103.2698663	
812.4518034	609.2877011	
826.4417516	22.572628	LQPFPQP / PFPQPQL / FPQPQLP
831.4172661	22.89144308	
844.4045131	80.94524546	PF-LQL
864.441843	26.81948687	
867.3964384	20.99783856	PF-LQL + Na
890.4452063	20.52522159	
916.3959416	77.04785826	
923.5039728	22.20680842	PFPQPQLP
953.4828984	22.97742947	
954.4710861	20.96283965	LQPFPQPQ / QPFPQPQL
975.5154337	283.5428792	
987.5167808	107.9209289	
989.5261766	27.1283928	FPQPQLPY
1051.556015	22.44249806	QPFPQPQLP
1056.563536	15.96003427	
1086.58173	148.6973953	PFPQPQLPY
1150.570084	119.9457797	
1214.62244	114.6187676	QPFPQPQLPY
1219.606092	30.01407262	
1221.575663	10.94488857	
1236.616914	26.94008778	QPFPQPQLPY + Na
1278.609292	13.24000232	
1327.68016	11.09550225	LQPFPQPQLPY
1334.653232	15.43848585	
1391.700247	21.5082215	
1427.766227	13.03959043	
1455.751791	44.2855354	QLQPFPQPQLPY
1462.703504	9.563417287	
1477.739421	23.13084511	QLQPFPQPQLPY + Na
1568.820967	41.4356327	LQLQPFPQPQLPY
1575.771027	12.31257754	
1590.81785	56.87305011	LQLQPFPQPQLPY + Na
1632.846558	12.86595128	
1689.788025	20.79615407	
1898.906421	123.7180394	PF-LQLQPFPQPQLP + Na
1900.917521	49.81217064	PF-LQLQPFPQPQLP (open) + Na
1934.922471	36.66900563	
2039.980143	1341.884659	PF-LQLQPFPQPQLPY
2042.035384	158.5627459	PF-LQLQPFPQPQLPY
2045.942412	48.70076149	
2062.006485	2751.119086	PF-LQLQPFPQPQLPY + Na
2063.006137	4179.733016	PF-LQLQPFPQPQLPY (open) + Na
2079.028049	68.71813811	PF-LQLQPFPQPQLPY + K
2084.045616	296.6452011	
2086.074003	198.1809859	
2200.109737	23.36134747	

B

basal sample 1

m/z	area	
516.9655353	835.2569537	
552.3001799	644.3418318	
575.5033059	123.964294	
579.8564488	708.0814315	
607.6837862	229.6304333	
668.5903056	10.95963805	
739.3603833	17.99076392	
768.3823762	85.81262421	
916.4766263	51.22771309	
1218.71271	10.19787309	
1232.758432	13.48483502	
1606.80792	89.96995258	
1665.801033	193.1585032	PF-LQLQPFPQPQ
1735.816297	618.0775367	
1836.005117	29.33988561	
1949.981937	16.37100099	
2092.034154	31.33218481	
2191.03514	37.21979734	

basal sample 2

m/z	area
507.3042325	243.9508347
516.8467964	838.1907735
523.8379964	260.1268048
552.1967622	315.4209873
561.8454475	114.5295448
573.1114845	62.95829544
575.3768994	92.93440191
579.6821884	1478.879359
597.3712304	63.68579378
607.5605566	414.69084
613.3341496	43.37404149
640.4816889	132.8906093
667.2873769	115.1398949
668.5033229	113.7532096
675.2229293	35.76401162
724.373538	1580.202645
762.3989723	49.88307806
765.3562017	41.85468596
768.290051	121.0754609
781.4568551	3759.193815
803.4579096	46.23993325
809.4742553	153.7604574
820.4237527	98.32779107
825.4782054	158.054239
916.3702348	60.73250328
1218.642667	38.25954737
1251.610977	20.45238405
1310.617044	20.18037145
1594.82998	16.64761242
1606.775419	97.43347477
1665.797328	137.9550233
1735.821248	762.4017791
1757.834864	53.38338248
1835.984744	51.93962668
2092.052935	51.66281554
2191.074471	26.27072964

basal sample 3

m/z	area	
516.9582485	1716.991153	
534.2570141	98.02131013	
538.4168882	199.2445204	
552.3663017	257.2852135	
567.3999584	59.00719498	
573.2785514	107.8437626	
575.5176199	484.1890446	
579.9128051	286.9326991	
595.0161696	37.86016583	
597.3477307	50.26036476	LQLQP
607.7017282	98.87736681	
613.4632934	37.59649976	
640.6126212	49.98689731	
668.6250512	102.0438197	
696.6580938	23.98696682	
739.3739189	78.6431511	
757.4090268	18.52091369	
768.4184559	482.8386106	
775.4255252	18.46473712	
777.5197505	19.48320627	
860.4586552	20.12631057	
906.950578	15.82034201	
916.5275179	158.390088	
1218.897337	90.2230735	
1232.942934	39.60667435	
1251.936591	26.12250642	
1310.954691	40.91345502	
1595.338421	47.76477665	
1607.28214	273.0874497	
1629.327919	28.62137202	
1666.347419	442.3564189	
1736.426576	1556.827541	
1758.45009	160.1323172	
1836.690576	172.5650582	
1849.833512	60.51213611	
1916.624628	25.69635244	
1950.823561	35.4700036	
2093.004755	145.8583089	
2192.18299	47.54301676	
2205.072223	23.28205687	
