# Supplementary material for: Epithelial Transport of Immunogenic and Toxic Gliadin Peptides In Vitro
Source: PLoS One. 2014 Nov 21;9(11):e113932. doi: 10.1371/journal.pone.0113932 (PMC4240668; doi:10.1371/journal.pone.0113932)
Supplement: Table S2 — Fragments of P56–68 after 24 h incubation summarized in Figure 4 A. After incubation of the fluorescence labeled (PromoFluor-488, PF) P56–68 (PF-P56–68) at the apical side of the Caco-2 monolayer, the majority of P56–68 was cleaved into several fragments (A). Analysis of the basal media revealed translocation of some intact P56–68 as well as fragments (B). Analysis of the molecular masses was done by MALDI-TOF-MS. The detected molecular masses were assigned to the masses of P56–68 and fragments thereof. The experiment was repeated 3 times (sample 1, 2 and 3). (PDF) [file pone.0113932.s004.pdf]

24h incubation of Caco-2 cells with PF-p56-68

A

apical
sample 1

m/z	area	
552.2118172	723.0134726	
557.9920323	476.5281652	
559.9428294	995.4634617	
565.8659345	590.0157427	
579.8298134	1985.795818	
582.6740607	372.1589856	
587.595986	282.2177291	
603.4046197	1122.093951	PF-L
607.6636107	2000.265724	
618.3371001	328.7413275	
627.3457649	116.2836401	
649.2964108	160.1344539	
675.3496069	148.7940582	
679.3512428	1324.674993	
680.39019	351.7268419	
693.3511715	288.1539916	
697.4106453	287.8799153	
713.324149	283.3712608	PFPQPQ / QPFPQP
715.3402062	669.0820353	
721.3420802	117.8552132	
731.2729107	9803.759446	PF-LQ
743.3588811	557.3214612	
753.2713717	377.1287133	PF-LQ + Na
755.3144598	171.9503669	
768.3437356	106.5035675	
777.4260103	705.2102086	
790.4128017	585.6599901	
812.4212454	2822.454732	
826.4318022	496.0843248	LQPFPQP / PFPQPQL / FPQPQLP
828.3767007	319.291358	
844.3741986	1939.739513	PF-LQL
850.4283423	120.2075303	
890.4411017	252.4675786	
904.480079	154.0283997	
923.4987156	839.9353465	PFPQPQLP
954.4803181	249.1456526	
965.4858163	219.2107053	
972.4511435	1154.766073	PF-LQLQ
987.5018611	2415.582164	
1036.561156	344.0641788	
1051.566096	843.7150252	QPFPQPQLP
1056.560764	235.0459513	
1067.565353	103.180135	LQLQPFPQP
1069.501549	529.5110323	PF-LQLQP
1092.516514	118.6751928	PF-LQLQP + Na
1115.572607	653.1897668	
1131.598986	87.11458049	
1164.652438	582.6886785	LQPFPQPQLP
1186.618585	111.54279	LQPFPQPQLP + Na
1214.638384	91.13842116	QPFPQPQLPY
1216.578053	87.05536214	PF-LQLQPF
1228.656028	493.1561985	
1264.606018	107.9347035	
1292.716903	275.0532193	QLQPFPQPQLP
1297.674796	93.98509197	
1313.642296	134.9525654	PF-LQLQPFP
1335.618414	103.1781848	PF-LQLQPFP + Na
1390.725078	139.6245152	
1405.76454	78.9194594	LQLQPFPQPQLP
1412.716488	248.8577922	
1425.7311	97.99021397	
1427.74967	171.6562147	LQLQPFPQPQLP + Na
1441.695067	105.5012368	PF-LQLQPFPQ
1453.782888	70.85303097	
1518.855332	308.8301279	
1538.754024	107.2207533	PF-LQLQPFPQP
1560.739456	115.7380405	PF-LQLQPFPQP + Na
1665.738181	141.8188587	
1666.781821	626.3395936	PF-LQLQPFPQPQ
1688.779254	516.1059851	PF-LQLQPFPQPQ + Na
1690.797288	215.6698833	
1735.797681	280.3854398	
1779.842257	452.3282409	PF-LQLQPFPQPQL
1781.801253	257.3176603	PF-LQLQPFPQPQL
1802.87433	717.2862553	
1835.878828	193.6822185	
1857.822272	87.09974177	
1876.945254	1937.913604	PF-LQLQPFPQPQLP
1898.991491	2135.141825	PF-LQLQPFPQPQLP + Na
1899.940763	3688.578536	PF-LQLQPFPQPQLP (open) + Na
1915.971666	130.7689623	PF-LQLQPFPQPQLP + K
1921.966001	69.06821148	
2040.041925	250.2604317	PF-LQLQPFPQPQLPY
2062.094479	310.9432736	PF-LQLQPFPQPQLPY + Na
2063.050193	820.5939367	PF-LQLQPFPQPQLPY (open) + Na
2092.055136	89.89726247	
2191.064774	67.14416122	

apical
sample 2

m/z	area	
546.0347259	1337.724194	
557.8540062	502.32478	
559.8374914	1120.418592	
565.7787184	628.0773918	
582.6303843	412.4321711	
585.5660811	291.2137465	
587.5625451	428.1544436	
603.3908125	2012.035292	PF-L
627.3376076	311.1480715	
649.3113462	166.5281007	
675.360155	177.2451381	
679.3625042	1555.816169	
680.3944941	418.2198129	
693.3626592	479.0936224	
697.3926055	500.8943705	
713.3331787	376.7826188	QPFPQP / PFPQPQ
715.3452166	585.0692659	
721.3525789	179.4492027	
731.2810591	9853.723762	PF-LQ
743.3636326	436.3136309	
753.2812618	321.2692616	PF-LQ mit Na
755.3364216	170.287028	
768.354063	252.4754605	
777.4377589	373.355675	
790.4286341	583.7608325	
812.4307446	1621.393827	
826.4441052	494.042244	LQPFPQP / PFPQPQL / FPQPQLP
828.3785618	256.1948297	
844.3911503	2140.085539	PF-LQL
923.5113858	895.6367954	PFPQPQLP
954.4885705	263.921169	LQPFPQPQ / QPFPQPQL
965.5108304	159.296885	
972.4659426	934.2226264	PF-LQLQ
987.5127822	899.3346246	
1036.570022	141.4229135	
1051.575607	574.4874284	QPFPQPQLP
1056.549638	115.8169791	
1069.526281	539.2412669	PF-LQLQP
1115.578218	176.8515467	
1164.660622	311.7507979	LQPFPQPQLP
1216.605151	147.8232923	PF-LQLQPF
1228.652582	146.9665673	
1292.728502	127.9937238	QLQPFPQPQLP
1313.668729	153.9676679	PF-LQLQPFP
1336.59843	192.5019386	PF-LQLQPFP + Na
1405.806675	69.01746644	LQLQPFPQPQLP
1426.706234	138.5965565	
1428.700429	112.1198351	LQLQPFPQPQLP + Na
1441.714237	188.0945866	PF-LQLQPFPQ
1538.77118	222.2521279	PF-LQLQPFPQP
1560.70043	82.54614368	PF-LQLQPFPQP + Na
1666.802748	1078.410466	PF-LQLQPFPQPQ
1689.7766	401.1701926	PF-LQLQPFPQPQ + Na
1735.772171	182.4837743	
1763.862607	102.0851043	
1779.835505	1295.591322	PF-LQLQPFPQPQL
1802.838965	582.146596	PF-LQLQPFPQPQL + Na
1835.866211	151.504151	
1876.971899	4866.206077	PF-LQLQPFPQPQLP
1899.022066	1685.292079	PF-LQLQQPFPQPQLP + Na
1899.955711	3121.880827	PF-LQLQPFPQPQLP (open) + Na
1915.930815	126.6820094	PF-LQLQPFPQPQLP + K
2040.087469	512.1278885	PF-LQLQPFPQPQLPY
2063.033994	509.9686915	PF-LQLQPFPQPQLPY + Na
2092.050554	57.842434	

apical
sample 3

m/z	area	
546.0641988	1325.749807	
557.8796962	429.0326336	
559.8336172	1026.458639	
565.7485628	486.6215249	
582.6079836	466.7950509	
587.5645403	354.6963746	
603.3821205	2226.705505	PF-L
618.3348624	609.4521362	
627.3349448	220.7458666	
675.3709152	142.1323437	
679.3744687	1574.609878	
680.4184915	291.4032138	
693.3659767	410.2936664	
697.342245	283.9149111	
713.3489587	387.0151462	QPFPQP / PFPQPQ
715.3631819	488.8423989	
721.3585496	126.5818678	
731.2969498	10949.25691	PF-LQ
743.3896605	272.0877292	
753.300144	327.5959561	PF-LQ + Na
755.3397019	180.1487285	
760.5900811	131.7727491	
768.3655771	215.6530102	
777.4569075	395.3129208	
790.4275541	422.0330015	
812.4478341	1247.415641	
826.4595085	727.4346835	LQPFPQP / PFPQPQL / FPQPQLP
828.3961336	218.318813	
844.4009677	2016.888236	PF-LQL
916.4047878	131.3582075	
923.5266556	801.0381011	PFPQPQLP
954.4983177	284.4524114	
972.4724221	847.582387	PF-LQLQ
987.5301419	773.7088249	
1036.583672	131.90251	
1051.585153	817.8893875	QPFPQPQLP
1056.580836	103.3810545	
1069.519445	415.6395216	PF-LQLQP
1086.597651	99.58414701	PFPQPQLPY
1091.533344	89.99175663	PF-LQLQP + Na
1115.598085	148.0474255	
1164.667548	279.653612	LQPFPQPQLP
1214.63865	93.10300005	QPFPQPQLPY
1216.598005	122.488917	PF-LQLQPF
1228.67705	159.4834803	
1292.710853	76.20462259	QLQPFPQPQLP
1313.636253	122.9853341	PF-LQLQPFP
1336.630583	151.7646057	PF-LQLQPFP + Na
1426.744653	165.111287	
1441.705772	132.2887299	PF-LQLQPFPQ
1456.753277	60.16135934	
1538.756625	186.0218669	PF-LQLQPFPQP
1561.726595	155.7728104	PF-LQLQPFPFP + Na
1666.785215	1023.540125	PF-LQLQPFPQPQ
1688.784886	736.5328527	PF-LQLQPFPQPQ + Na
1690.803359	393.0166331	
1735.757779	143.5568979	
1779.849826	1187.634037	PF-LQLQPFPQPQL
1802.857923	939.2541796	PF-LQLQPFPQPQL + Na
1835.842922	115.7154863	
1876.95777	4534.140605	PF-LQLQPFPQPQLP
1879.04151	282.2303988	
1882.943859	134.4712662	
1898.990903	3281.47707	PF-LQLQPFPQPQLP + Na
1899.959206	4426.069051	PF-LQLQPFPQPQLP (open) + Na
1913.968554	102.2239717	
1915.951303	245.7073185	PF-LQLQPFPQPQLP + K
2040.039308	1153.34899	PF-LQLQPFPQPQLPY
2042.005892	148.0780111	PF-LQLQPFPQPQLPY
2062.0752	770.5388621	PF-LQLQPFPQPQLPY + Na
2063.039334	1552.732517	PF-LQLQPFPQPQLPY (open) + Na
2092.006212	79.07739576	

B

basal
sample 1

m/z	area	
516.4001714	1708.746295	
533.6355614	440.3585256	
537.8786214	5236.806613	
553.4226394	221.1770428	
555.1783492	753.2578906	
572.8078737	256.8422315	
574.9765777	164.8450803	
576.7769075	226.3056675	
594.5046419	1274.605341	
596.7709911	390.6165687	LQLQP
610.3756162	103.0255681	
616.3880018	1322.806649	QPFPQ / FPQPQ
632.3264708	256.8350797	
638.3529879	1537.018972	QPFPQ / FPQPQ + Na
654.3234563	529.7913589	QPFPQ / FPQPQ + K
660.3440801	687.5585158	
674.632597	319.7017481	
676.3292894	321.9367597	
682.3499954	181.8306315	
698.3408627	108.5326553	
730.3827247	108.9661041	PF-LQ
730.925369	92.01795409	PF-LQ
738.9504024	275.5199056	
752.4170533	128.9979937	PF-LQ + Na
767.9982132	1009.350678	
774.4229413	153.1902383	
775.0087448	121.7023682	
792.4082226	193.4257956	
812.1295395	466.7062028	
814.4276579	393.994061	
830.429825	101.8041887	
836.4541365	496.4759208	
852.4563313	213.0902415	
858.4778372	379.372996	
874.4432888	300.701658	
880.500705	297.1539352	
896.4725498	259.5514327	
902.5188417	155.2274234	
916.1796876	612.2723082	
918.4939356	177.2696792	
919.1584973	91.82816474	
934.1825384	212.5503412	
971.4099557	107.200541	PF-LQLQ
987.3737852	131.8606021	
1012.527697	125.2045286	
1034.517599	156.4844732	
1076.49037	203.6897122	
1098.493275	159.9658266	
1218.609151	205.2466485	
1222.672347	77.24326311	
1251.611217	92.6136281	
1297.598758	76.08472021	
1311.622024	323.6951305	
1325.645765	407.6517642	
1339.673481	223.1383933	
1594.878662	134.8580523	
1606.818833	134.7539533	
1628.819924	235.5367182	
1665.830477	177.5274194	PF-LQLQPFPQPQ
1735.855977	490.6348688	
1739.83627	109.7710617	
1757.845472	1033.359762	
1779.841214	178.8427944	PF-LQLQPFPQPQL
1835.999328	225.481604	
1899.020566	94.33384808	PF-LQLQPFPQPQLP + Na
2092.026461	182.7922684	
2113.993122	95.51228723	

basal
sample 2

m/z	area	
516.5980532	1001.929427	
533.9872327	957.8904805	
538.0476567	1056.068441	
555.5455796	693.1332495	
573.088192	410.2290888	
575.3219946	296.2070673	
587.4235949	376.2429275	
594.9033418	1370.451106	
597.2008976	364.5715088	LQLQP
602.355979	140.1943926	PF-L
603.3217161	182.8220158	PF-L
613.3568318	218.6311584	
616.8052301	797.1480676	PQLPY
632.7505789	78.10557446	
638.7589496	578.5585936	QPFPQ / FPQPQ
639.2118782	261.2321241	
653.2354697	514.6999061	
660.7416008	154.0820034	
675.1373752	599.2225792	
679.2814478	117.5537716	
715.3068756	168.640746	
731.2409941	876.7449782	PF-LQ
739.2807831	171.1254404	
743.3159975	155.4561664	
752.7812481	111.2352331	
753.2386426	300.1808365	PF-LQ + Na
755.2827051	97.14729844	
768.3239468	600.668471	
777.3409146	145.1247342	
792.7318297	144.4422999	
812.4031407	1124.555698	
836.7483338	107.7134601	
916.409113	222.0250101	
987.5301694	308.7606735	
1218.653051	410.5772575	
1251.671118	106.282199	
1297.632366	87.43409174	
1311.642375	404.6168343	
1325.655805	497.2425444	
1339.682224	426.0044195	
1353.685454	125.0843689	
1594.835613	289.2433488	
1606.77663	662.2443078	
1628.811117	498.2773096	
1665.781524	2168.252469	
1687.76388	151.9214331	
1735.838975	3546.712671	
1739.805097	398.2509739	
1757.853508	2605.238416	
1779.860557	337.0098787	PF-LQLQPFPQPQL
1835.974011	1006.136202	
1898.980101	348.465086	PF-LQLQPFPQPQLP + Na
1949.977467	323.5816816	
2092.086976	562.4759802	
2114.076775	201.9418951	
2191.061048	791.329243	
2204.054656	194.6682617	

basal
sample 3

m/z	area	
516.784402	674.0622854	
552.2226437	787.6689945	
575.4166729	141.9632756	
579.834632	1204.646778	
588.5471406	139.2483312	
603.3584514	238.8530391	PF-L
607.6621362	736.1745523	
613.4383484	153.1215218	
618.3709666	95.26005826	
640.5648897	532.2585212	
654.56853	75.32659845	
668.5790393	1018.470947	
679.3409164	104.6256225	
694.5985834	130.1222061	
696.6173471	476.1698112	
731.2835972	1078.380506	PF-LQ
739.3261175	206.916256	
753.2855561	66.63123307	PF-LQ + Na
768.3557167	153.7470282	
812.436196	190.3903096	
826.4470357	68.96729365	LQPFPQP / PFPQPQL / FPQPQLP
844.4085011	152.0133519	PF-LQL
878.8419124	94.85943961	
892.8412133	57.34796795	
906.8758263	228.1920965	
916.4195449	123.3253156	
932.9015377	73.68264069	
934.9107933	168.402933	
987.5410448	59.34180102	
1069.553698	66.60180001	PF-LQLQP
1091.543619	65.60258947	PF-LQLQP + Na
1218.634037	217.1714837	
1251.636893	140.3042485	
1311.622949	162.4308837	
1325.657867	173.4705094	
1339.660248	194.5928643	
1353.683613	60.53651507	
1381.731678	58.02884815	
1427.754731	71.86411721	
1594.811297	227.7883492	
1606.774592	602.0194767	
1628.757948	91.10533266	
1665.766356	1516.791039	
1735.820953	3554.343722	
1757.821886	631.3871631	
1835.93525	1130.960342	
1849.111987	237.9471595	
1876.987387	104.3903468	PF-LQLQPFPQPQLP
1899.983945	187.694936	PF-LQLQPFPQPQLP + Na
1949.948588	261.2480928	
2063.041924	135.9216907	PF-LQLQPFPQPQLPY + Na
2092.057888	1105.659807	
2191.080374	625.5323015	
2204.060024	135.2336181	
