# Supplementary material for: Epithelial Transport of Immunogenic and Toxic Gliadin Peptides In Vitro
Source: PLoS One. 2014 Nov 21;9(11):e113932. doi: 10.1371/journal.pone.0113932 (PMC4240668; doi:10.1371/journal.pone.0113932)
Supplement: Table S3 — Fragments of P31–43 after 3 h incubation summarized in Figure 4 B. After incubation of the fluorescence labeled (PromoFluor-488, PF) P31–43 (PF-P31–43) at the apical side of the Caco-2 monolayer, P31–43 was partially cleaved into several fragments (A). Analysis of the basal media revealed translocation of some intact P31–43 as well as fragments (B). Analysis of the molecular masses was done by MALDI-TOF-MS. The detected molecular masses were assigned to the masses of P31-43 and fragments thereof. The experiment was repeated 3 times (sample 1, 2 and 3). (PDF) [file pone.0113932.s005.pdf]

3h incubation of Caco-2 cells with PF-p31-43

A

apical sample 1

m/z	area	
513.4634311	1.983496602	
517.0380988	23.25073229	
530.2944459	9.838972066	
567.0311305	11.3470109	
586.6418035	4.804227593	
607.7789114	3.105393625	
696.37945	4.337287398	
713.3758899	29.57998325	QQPFPP / QPFPPQ / FPPQQP / PFPPQQ
729.4085079	42.57195837	PPQQPY
810.4418871	4.302190478	PF-LGQ + Na
811.4576572	17.1551564	PF-LGQ (open) + Na
876.4251951	4.742797808	FPPQQPY
916.3748262	6.186456415	PF-LGQQ
973.501952	8.074334601	PFPPQQPY
974.511912	4.358122693	
1229.550541	5.716470454	QQPFPPQQPY
1251.610507	8.06299445	QQPFPPQQPY + Na
1267.622506	3.409699352	LGQQQPFPPQQ / QQPFPPQQPY + K
1364.706618	7.888658307	LGQQQPFPPQQP
1390.666867	8.919140605	
1412.713297	4.556462049	
1414.671348	14.08637698	GQQQPFPPQQPY
1492.810204	43.72794979	
1527.783201	18.48690886	LGQQQPFPPQQPY
1553.758075	13.80393563	
1575.761703	5.737776656	
1655.892815	147.512611	
1835.869221	13.87495456	PF-LGQQQPFPPQQP
1857.889294	12.03858463	PF-LGQQQPFPPQQP + Na
1998.965851	301.7270275	PF-LGQQQPFPPQQPY
2021.020275	212.2766532	PF-LGQQQPFPPQQPY + Na
2023.036975	80.64608512	

apical sample 2

m/z	area	
507.5549103	155.3289077	
517.0838456	229.3227935	
575.6086945	60.11196378	
579.8396672	185.9571191	
586.686771	50.48113318	
626.2731938	11.05237649	
659.3301534	13.23451599	PF-LG
660.3333225	15.28180859	PF-LG
675.3970694	10.89234053	
692.3645742	567.3055435	LGQQQP + Na
694.3878328	159.240984	
696.4341779	80.19672627	
713.406616	51.81709998	QQPFPP / QPFPPQ / FPPQQP / PFPPQQ
724.5336715	94.97697876	
729.4205749	100.7819766	PPQQPY
768.4064531	73.62961866	PPQQPY + K
781.580568	1196.5019	
788.3514174	20.23321964	PF-LGQ
809.5686943	22.91885781	
811.4813871	113.7305548	PF-LGQ (open) + Na
825.5715441	40.25180602	
876.4467212	29.91930178	FPPQQPY
916.406178	91.90880783	PF-LGQQQ
973.5055479	34.08607706	
974.5237151	37.54496003	
1229.576032	10.94758728	QQPFPPQQPY
1267.675609	16.05209013	LGQQQPFPPQQ / QQPFPPQQPY + Na
1341.90797	5.884303563	
1357.637817	9.435250588	QQQPFPPQQPY
1364.713363	6.265420855	LGQQQPFPPQQP
1414.672951	15.59132317	GQQQPFPPQQPY
1492.8031	48.35395349	
1527.777459	30.22339102	LGQQQPFPPQQPY
1553.743378	17.27561923	
1655.879607	394.6919105	
1835.863269	11.16108439	PF-LGQQQPFPPQQP
1870.863359	7.858596656	
1998.970867	471.1974259	PF-LGQQQPFPPQQPY
2021.026988	72.43106293	PF-LGQQQPFPPQQPY + Na
2023.055677	42.19732605	

apical sample 3

m/z	area	
566.973921	238.7246325	
579.9384042	38.31717018	
586.6750268	63.64793253	
607.7437399	57.7235133	
674.3349231	20.97208284	
683.4121147	21.640123	PF-LG
696.3877934	92.20029261	
699.3828805	13.55242829	
713.3897588	381.8362971	QQPFPP / QPFPPQ / FPPQQP / PFPPQQ
729.3860824	883.2581471	PPQQPY
735.3862798	25.66462387	QQPFPP + Na / QPFPPQ +Na / FPPQQP + Na / PFPPQQ + Na
810.4337836	41.87614773	PF-LGQ + Na
811.4557999	159.9613986	PF-LGQ (open) + Na
841.4362149	23.1995191	QQPFPPQ / QQQPFPP / QPFPPQQ / QQPFPPQ
876.4466124	177.8674824	FPPQQPY
916.3944269	18.49979733	PF-LGQQ
938.4844059	35.37719294	QPFPPQQP / PF-LGQQ + Na
973.4958766	84.8502062	PFPPQQPY
974.5059454	62.73466856	
1026.479039	9.779276057	GQQQPFPPQ
1036.565474	12.93674918	
1101.542716	22.36271266	QPFPPQQPY
1139.597422	15.27547797	QPFPPQQPY + K
1187.596853	10.08643487	
1194.577167	11.26325594	QQQPFPPQQP
1229.59962	76.7101412	QQPFPPQQPY
1251.604583	41.26894173	QQPFPPQQPY + Na
1267.681127	120.8596215	LGQQQPFPPQQ / QQPFPPQQPY + K
1293.613069	19.58105755	
1357.645995	25.27928091	QQQPFPPQQPY
1364.702722	57.31449894	LGQQQPFPPQQP
1390.688751	63.3743992	
1395.727181	18.52430299	
1412.718661	70.67468494	
1414.695898	169.4015104	GQQQPFPPQQPY
1436.679523	21.64886551	GQQQPFPPQQPY + Na
1478.710904	18.52508608	
1492.798285	609.9375067	
1514.786755	22.98230692	
1527.783884	308.6966267	LGQQQPFPPQQPY
1549.753574	73.04795764	LGQQQPFPPQQPY + Na
1553.781287	224.6011998	
1575.782712	393.6792989	
1591.764828	20.95832778	
1655.883258	3035.704672	
1677.891373	234.392735	
1857.869234	80.8096981	PF-LGQQQPFPPQQP + Na
1998.970907	307.0117042	PF-LGQQQPFPPQQPY
2020.986308	1528.241704	PF-LGQQQPFPPQQPY + Na
2023.020812	576.3932076	
2037.001212	91.23799054	PF-LGQQQPFPPQQPY + K
2043.016696	25.28494208	

B

basal sample 1

m/z	area	
502.477877	209.8503218	
516.9413716	1698.892943	
552.3353768	218.4371241	
575.4709526	296.0889609	
579.8941308	328.8114087	
607.6875543	352.634646	
613.4563173	27.85779565	
616.2888885	35.5819093	
640.6003011	19.00427894	
668.603482	15.97459554	
713.345626	24.12595475	QQPFPP / QPFPPQ / FPPQQP / PFPPQQ
729.3710364	96.44127334	PPQQPY
739.3208864	64.89790802	
768.3428582	185.5655902	PPQQPY + K
811.441964	26.31148694	PF-LGQ (open) + Na
916.3865855	217.2565192	PF-LGQQ
973.5221582	18.16307816	PFPPQQPY
1218.605078	34.25686002	
1251.629894	23.12642995	GQQQPFPPQQP
1492.853301	36.07298887	
1527.823694	30.98179082	LGQQQPFPPQQPY
1553.816241	22.26691616	
1594.780164	26.87497342	
1606.759308	227.4156457	
1655.946328	136.4761571	
1665.767791	474.3306193	
1735.805797	1473.627368	
1757.786253	34.09705	
1835.945026	199.5247908	PF-LGQQQPFPPQQP
1949.979156	34.29655899	
1998.979951	301.5865233	PF-LGQQQPFPPQQPY
2021.970927	20.01674685	PF-LGQQQPFPPQQPY + Na
2092.076122	89.78513137	
2191.147701	96.42048771	
2204.063498	34.98771374	

basal sample 2

m/z	area	
516.7802273	492.151887	
552.1411287	719.4589226	
561.7361234	92.80418343	
579.6823543	1666.090517	
597.3367285	67.40061097	
607.5318235	623.3217749	
613.3045706	58.23496787	
640.4306223	39.42252802	
667.2425241	60.87064613	
668.4511986	27.6999815	
685.3887854	78.122602	
703.3800649	29.24758121	
724.325485	714.7667667	
731.1426107	32.21888022	
760.4306618	26.87754485	
762.3585186	36.53533809	
765.3200848	39.41213197	
768.3129172	51.02803596	
781.4133489	1015.971478	
809.4240279	83.44597238	
825.4201459	169.9107492	
962.6546106	22.17544134	
990.6786203	23.66610753	
1046.521438	26.07329648	
1218.594675	15.23108512	
1568.90991	17.09543524	
1606.767943	178.2959511	
1665.777415	451.7988459	
1735.816894	1673.167565	
1757.867699	23.89210267	
1835.965535	69.76796171	PF-LGQQQPFPPQQP
1949.974664	23.02970662	
1998.981629	16.48738546	PF-LGQQQPFPPQQPY
2040.050869	97.55219088	
2092.074344	39.00330199	
2191.127414	92.61304829	
2204.089348	35.42113462	
2271.022568	13.71687344	

basal sample 3

m/z	area	
517.0157353	1486.235122	
523.9664144	274.8459486	
526.0286311	228.9314086	
530.233583	116.4584109	
538.404946	82.72873377	
552.4033647	1021.245228	
573.2659551	63.62525027	LGQQQ
575.5590168	526.8167366	
579.9411715	1799.4915	
607.7250843	1366.254164	
613.5202944	31.75561563	
739.3761828	52.99157995	
749.4856218	39.06705692	
768.4022766	515.074665	
777.4950722	70.26563187	
805.5262041	51.30268897	
860.3974564	17.75658251	
916.4668534	123.68233	PF-LGQQ
1218.702912	45.01965657	
1232.770823	23.76526717	QQQPFPPQQP + K
1310.661534	22.16659233	
1594.874492	18.17172132	
1606.786806	173.8113508	
1665.800392	291.8745824	
1735.816709	972.3975419	
1757.805673	44.95757218	
1835.967306	75.57342494	PF-LGQQQPFPPQQP
2092.028951	52.11277076	
2191.098917	33.22670923	
