# Supplementary material for: Epithelial Transport of Immunogenic and Toxic Gliadin Peptides In Vitro
Source: PLoS One. 2014 Nov 21;9(11):e113932. doi: 10.1371/journal.pone.0113932 (PMC4240668; doi:10.1371/journal.pone.0113932)
Supplement: Table S4 — Fragments of P31–43 after 24 h incubation summarized in Figure 4 B. After incubation of the fluorescence labeled (PromoFluor-488, PF) P31–43 (PF-P31–43) at the apical side of the Caco-2 monolayer, the majority of P31–43 was cleaved into several fragments (A). Analysis of the basal media revealed translocation of some intact P31–43 as well as fragments (B). Analysis of the molecular masses was done by MALDI-TOF-MS. The detected molecular masses were assigned to the masses of P31-43 and fragments thereof. The experiment was repeated 3 times (sample 1, 2 and 3). (PDF) [file pone.0113932.s006.pdf]

24h incubation of Caco-2 cells with PF-p31-43

A

apical
sample 1

m/z	area	
516.9628052	20.09756278	
558.032109	51.5351492	
566.9862742	297.8230468	
575.5160695	12.07602739	
586.6717764	78.90982273	
603.4517495	21.58152384	PF-L
618.3943979	14.77733987	
660.2831145	90.80444088	PF-LG
683.4062185	18.43678691	PF-LG + Na
696.3683731	8.923141822	
713.3490316	201.6764417	QQPFPP / QPFPPQ / FPPQQP / PFPPQQ
729.3789504	13.41618019	PPQQPY
735.3442334	13.28185214	QQPFPP +Na / QPFPPQ +Na / FPPQQP + Na / PFPPQQ + Na
768.3923553	7.399442411	
788.3641775	5.667916682	PF-LGQ
810.3990312	44.6057824	PF-LGQ + Na
811.4191693	175.0266571	PF-LGQ (open) + Na
841.4621438	12.28482106	QQPFPPQ / QQQPFPP / QPFPPQQ / QQPFPPQ
916.408658	14.53607461	PF-LGQQQ
938.4944891	12.65063393	QPFPPQQP / PF-LGQQ + Na
939.5066885	43.21605316	PF-LGQQQ (open) + Na
969.4692201	4.712886874	QQQPFPPQ / QQPFPPQQ
1026.489559	14.9328964	GQQQPFPPQ
1036.554932	25.60612934	
1124.595257	5.71003159	
1139.603343	7.778105559	LGQQQPFPPQ
1141.486222	21.91259989	PF-LGQQQP
1187.573536	26.67859792	
1194.632049	9.684766391	QQQPFPPQQP
1251.663451	23.8466675	GQQQPFPPQQP
1364.755692	11.95225874	LGQQQPFPPQQP
1390.726688	44.87872789	
1412.685292	46.15939337	
1610.691832	83.0107539	PF-LGQQQPFPPQ
1632.733645	71.63188462	PF-LGQQQPFPPQ + Na
1835.853724	764.5484038	PF-LGQQQPFPPQQP
1857.923631	1527.479747	PF-LGQQQPFPPQQP + Na
1859.955967	501.055482	
1874.927977	23.0557223	PF-LGQQQPFPPQQP + K
2021.061087	36.88688344	PF-LGQQQPFPPQQPY + Na

apical
sample 2

m/z	area	
534.2113914	54.17933723	QQPY
557.9542941	360.7313752	QQPY + Na
559.8857056	57.83651756	
566.9200858	938.7413112	
573.2294923	38.15898718	LGQQQ / QQPY + K
585.6205116	66.62150968	
586.617564	148.0400641	
602.4565035	36.29956121	PF-L
603.4183481	155.1711528	PF-L
660.3098172	787.5874295	PF-LG
682.284634	93.3809133	
683.3963361	56.32407542	PF-LG + Na
684.2972091	26.44280465	
696.3752964	19.51371599	
713.3992177	510.0860711	QQPFPP / QPFPPQ / FPPQQP / PFPPQQ
718.3939236	30.35459205	
720.3758544	24.69979604	
729.4167536	94.70003722	PPQQPY
735.3904072	114.1231155	QQPFPP +Na / QPFPPQ +Na / FPPQQP +Na / PFPPQQ + Na
787.3885748	24.13625976	PF-LGQ
788.3740549	37.76629772	PF-LGQ
810.46409	230.6984735	PF-LGQ + Na
811.4875028	421.4384856	PF-LGQ (open) + Na
841.4554467	20.63144503	QQPFPPQ / QQQPFPP / QQPFPPQ / QPFPPQQ
846.4421355	25.33205958	
905.482411	46.96112673	
915.4378299	24.70424597	PF-LGQQ
916.4251624	66.19977487	PF-LGQQ
938.5093738	94.73681147	QPFPPQQP / PF-LGQQ + Na
939.5287481	115.4426846	
973.5171652	20.77554179	PFPPQQPY
974.5169919	29.22967454	
1026.495903	59.586076	GQQQPFPPQ
1033.510066	23.19775449	LGQQQPFPP + Na
1036.601555	64.38932358	
1066.56811	44.22283367	PF-LGQQQ + Na
1071.54479	25.37347896	
1088.562451	25.80898521	QQPFPPQQP + Na
1090.569598	24.2731137	
1130.580783	49.5138605	
1141.533196	194.076717	PF-LGQQQP
1163.565547	156.9772854	PF-LGQQQP + Na
1187.640546	340.4104455	
1251.653927	38.84070069	GQQQPFPPQQP
1258.650046	35.17331017	
1270.6006	35.26745769	
1273.650868	41.56909662	GQQQPFPPQQP + Na
1315.685993	69.45119343	
1364.721323	42.0753175	LGQQQPFPPQQP
1384.716992	28.2403574	PF-LGQQQPFP
1386.720311	74.66432494	LGQQQPFPPQQP + Na / PF-LGQQQPFP (open)
1390.72346	116.4135465	
1412.763327	662.6127512	
1414.752871	84.79560973	GQQQPFPPQQPY
1482.710107	61.28107321	PF-LGQQQPFPP
1504.71788	91.01085281	PF-LGQQQPFPP + Na
1506.726495	46.76400373	
1610.761839	400.2005772	PF-LGQQQPFPPQ
1632.780467	659.2021904	PF-LGQQQPFPPQQ
1634.805633	320.5614018	
1729.840677	68.66505387	
1738.825965	89.08244221	PF-LGQQQPFPPQQ
1740.388367	119.2387879	
1761.838611	121.9209108	PF-LGQQQPFPPQQ + Na
1835.893631	2111.947098	PF-GQQQPFPPQQP
1837.972736	206.0896296	
1857.921048	2890.217714	PF-LGQQQPFPPQQP + Na
1858.914861	3573.329344	PF-LGQQQPFPPQQP (open) + Na
1874.924308	76.73175411	PF-LGQQQPFPPQQP + K
1879.915986	56.0256795	
1881.930119	59.41207824	
1999.001394	164.1191795	PF-LGQQQPFPPQQPY
2021.026703	205.1608789	PF-LGQQQPFPPQQPY + Na
2022.02519	343.8635039	PF-LGQQQPFPPQQPY (open) + Na

apical
sample 3

m/z	area	
507.4371068	556.1098132	
558.0020477	817.7837876	
559.991665	103.70434	
566.9641213	2493.145597	
575.5484664	192.2815619	
586.6434637	1395.120287	
603.4217913	371.7197694	PF-L
616.4118788	44.60070734	
618.353475	151.129011	
660.2516548	1654.469559	PF-LG
683.3696113	388.2628583	PF-LG + Na
696.3440526	121.1033299	
713.3394278	2216.749375	QQPFPP / QPFPPQ / FPPQQP / PFPPQQ
718.3504453	65.50755188	
729.3488571	147.8633551	PPQQPY
735.3461954	155.8126291	QQPFPP +Na / QPFPPQ +Na / FPPQQP +Na / PFPPQQ + Na
760.5851466	50.38547751	
768.3603956	105.9380829	
788.319405	85.71471079	PF-LGQ
810.3997678	372.5756828	PF-LGQ + Na
811.4333481	2065.10033	PF-LGQ (open) + Na
841.4321338	209.2742478	QQPFPPQ / QQQPFPP / QQPFPPQ / QPFPPQQ
905.4372997	59.3372491	
916.3899145	210.9170344	PF-LGQQ
938.4768501	117.8654134	QPFPPQQP / PF-LGQQ + Na
939.5107697	578.9811654	PF-LGQQ (open) + Na
969.5047652	74.69126742	QQQPFPPQ / QQPFPPQQ
974.4954152	43.36292346	
1026.492215	146.7555649	GQQQPFPPQ
1036.574879	257.3236727	
1066.547896	220.5894762	PF-LGQQQ + Na
1124.584174	61.32783423	
1130.560707	44.46163189	
1139.600931	100.3385116	LGQQQPFPPQ
1141.510032	315.6435987	PF-LGQQQP
1164.63835	134.8789464	PF-LGQQQP + Na
1187.61697	436.172225	
1194.624411	114.1423351	QQQPFPPQQP
1251.644336	183.3039069	GQQQPFPPQQP
1315.66153	57.72287	
1364.720803	124.0028223	LGQQQPFPPQQP
1390.730209	380.3384597	
1412.739352	653.2443166	
1482.708099	106.5326102	PF-LGQQQPFPP
1492.841942	65.55343585	
1504.708776	70.69438717	PF-LGQQQPFPP + Na
1610.748801	603.8265479	PF-LGQQQPFPPQ
1632.73533	490.3175961	PF-LGQQQPFPPQ + Na
1634.777934	83.65351663	
1730.805901	73.08393941	
1760.77364	88.02301904	PF-LGQQQPFPPQQ + Na
1835.873857	3326.061776	PF-LGQQQPFPPQQP
1858.007169	2263.4905	PF-LGQQQPFPPQQP + Na
1858.895498	3648.391602	PF-LGQQQPFPPQQP (open) + Na
1874.905209	219.6951025	PF-LGQQQPFPPQQP + K
1999.010263	108.8148455	PF-LGQQQPFPPQQPY
2020.033254	59.42923774	PF-LGQQQPFPPQQPY + Na
2022.055181	192.5148467	PF-LGQQQPFPPQQPY (open) + Na

B

basal
sample 1

m/z	area	
502.5771718	152.4784298	
512.8531417	141.1150521	
517.1101292	1230.607246	
518.8713437	32.21330818	
522.1198432	45.13073105	
523.9859821	289.6229109	
526.0275677	117.0541848	
530.3122038	139.7356742	
534.2304731	72.86337096	QQPY
538.4707488	52.08408477	
545.3583174	37.26733228	
567.4563644	90.6687324	
573.3047956	73.00496316	LGQQQ
575.5984256	134.7406466	
579.9622668	85.06470595	
607.7546298	19.65009841	
640.6632302	12.95332631	
654.3315228	11.1787439	PQQPY + Na
660.3289554	26.33760348	PF-LG
668.657591	17.62672068	
713.4096564	15.69642794	QQPFPP / QPFPPQ / FPPQQP / PFPPQQ
739.4174258	37.13010624	
757.4131353	13.33970237	
768.4314962	102.4810919	
775.4264606	16.92452752	
860.4427847	12.52515379	
916.4676895	128.2260035	PF-LGQQQ
1035.497883	9.580862673	
1218.648553	12.69541213	
1251.664683	12.07848699	GQQQPFPPQQP
1311.647519	23.93597061	
1325.678612	21.84026047	
1339.670579	19.58837182	
1594.809445	7.191774718	
1606.76712	53.97044012	
1665.7942	129.8567035	
1735.813724	415.1558904	
1757.819371	29.51704622	
1835.915116	83.9306396	PF-LGQQQPFPPQQP
1857.92788	10.08442086	PF-LGQQQPFPPQQP + Na
1859.932083	8.842451495	
2092.048474	24.18886128	
2191.139796	24.23480762	

basal
sample 2

m/z	area	
501.31332	672.2462147	
516.8968811	1614.964755	
546.0372379	326.3183141	
552.2691386	2563.974413	
557.8605789	933.7767346	
559.8640195	145.2188894	
566.8860703	1064.89693	
568.7623825	151.5754823	
575.4964517	533.9667688	
579.8769841	1963.166333	
585.6111278	108.1916182	
586.5843727	159.5214715	
587.499187	304.510877	
603.4161104	210.097457	PF-L
607.703493	475.7046164	
613.4800194	117.3708254	
616.298834	138.734052	
660.2797843	1129.461775	PF-LG
713.3676378	331.3729798	QQPFPP / QPFPPQ / FPPQQP / PFPPQQ
729.3704214	130.7824539	PPQQPY
739.3434892	215.8692532	
768.3807876	286.4377079	PPQQPY + K
810.4300191	115.6572655	PF-LGQ + Na
811.4601383	337.4738715	PF-LGQ (open) + Na
916.4159954	184.253007	PF-LGQQ
938.5041247	76.49068189	QPFPPQQP / PF-LGQQ + Na
1036.556448	96.23849375	
1066.578338	61.61137817	PF-LGQQQ + Na
1141.565255	118.3984442	PF-LGQQQP
1194.625172	78.9054078	QQQPFPPQQP
1218.60486	141.9198773	
1251.651117	111.3836373	
1310.60248	194.8784978	
1364.736316	67.46558598	LGQQQPFPPQQP
1390.727094	84.66530298	
1594.756489	217.6237545	
1606.760487	672.5359392	
1610.763112	157.0378213	PF-LGQQQPFPPQ
1665.752171	3071.336514	
1735.838076	3114.967889	
1740.662357	140.5294966	
1757.732661	205.1396755	
1835.915765	2356.24498	PF-LGQQQPFPPQQP
1849.196394	150.7181682	
1857.782683	189.11744	PF-LGQQQPFPPQQP + Na
1859.871838	81.11894691	
1949.968699	415.6263637	
1998.983848	89.16816358	PF-LGQQQPFPPQQPY
2020.916053	73.11028516	PF-LGQQQPFPPQQPY + Na
2092.089361	531.3834003	
2191.13057	1354.662181	
2204.16602	141.8084291	

basal
sample 3

m/z	area	
516.8706164	1162.424264	
518.752787	109.7439374	
530.1441872	195.548868	
552.2919053	457.8299936	
557.9230729	100.1029514	
575.49725	563.5586029	
579.9355627	909.4320202	
587.5282915	124.214246	
603.4139876	49.2943266	PF-L
607.7497163	717.2084224	
613.5051842	44.01183772	
660.3030417	266.4117772	PF-LG
675.282141	50.85033289	
713.3845293	110.4786982	QQPFPP / QPFPPQ / FPPQQP / PFPPQQ
739.3770237	239.8514735	
757.4132784	77.24502137	
768.4011676	1086.188017	
775.4061386	26.47475776	
811.4820442	64.89161492	PF-LGQ (open) + Na
860.4193191	32.28182049	
916.4409894	178.2799848	PF-LGQQ
1218.652811	65.80786012	
1251.659185	29.40577071	
1310.654524	31.64137976	
1594.806599	75.58312828	
1606.745601	104.7365938	
1665.769615	341.1370674	
1735.789697	533.5885548	
1835.932594	346.0631936	PF-LGQQQPFPPQQP
1949.957003	38.87601806	
2092.092839	114.9106032	
2191.086258	104.7643251	
